# Silencing of *SlPL*, which encodes a pectate lyase in tomato, confers enhanced fruit firmness, prolonged shelf‐life and reduced susceptibility to grey mould

**DOI:** 10.1111/pbi.12737

**Published:** 2017-05-16

**Authors:** Lu Yang, Wei Huang, Fangjie Xiong, Zhiqiang Xian, Deding Su, Maozhi Ren, Zhengguo Li

**Affiliations:** ^1^ School of Life Sciences Chongqing University Chongqing China

**Keywords:** pectate lyase, *SlPL*, RNA interference, fruit shelf‐life, pathogen resistance, tomato

## Abstract

Pectate lyase genes have been documented as excellent candidates for improvement of fruit firmness. However, implementation of pectate lyase in regulating fruit postharvest deterioration has not been fully explored. In this report, 22 individual pectate lyase genes in tomato were identified, and one pectate lyase gene *SlPL* (Solyc03g111690) showed dominant expression during fruit maturation. RNA interference of *SlPL* resulted in enhanced fruit firmness and changes in pericarp cells. More importantly, the *SlPL*‐RNAi fruit demonstrated greater antirotting and pathogen‐resisting ability. Compared to wild‐type, *SlPL*‐RNAi fruit had higher levels of cellulose and hemicellulose, whereas the level of water‐soluble pectin was lower. Consistent with this, the activities of peroxidase, superoxide dismutase and catalase were higher in *SlPL*‐RNAi fruit, and the malondialdehyde concentration was lower. RNA‐Seq results showed large amounts of differentially expressed genes involved in hormone signalling, cell wall modification, oxidative stress and pathogen resistance. Collectively, these data demonstrate that pectate lyase plays an important role in both fruit softening and pathogen resistance. This may advance knowledge of postharvest fruit preservation in tomato and other fleshy fruit.

## Introduction

Postharvest deterioration is one of the biggest challenges for vegetable and fruit industries (Meli *et al*., 2010). The main determinant for the postharvest deterioration of fruit is the rate of softening, which limits fruit shelf‐life and affects wastage, postharvest pathogen‐induced infection and frequency of harvest (Brummell and Harpster, 2001). Hence, engineering fruit goods with better resistance to postharvest losses and diseases possesses potential value. Fruit softening is a complicated process regulated by a multitude of factors, among which cell wall modifying enzymes are considered to be the main contributors (Lunn *et al*., 2013). The plant cell wall is a highly complex matrix containing hemicellulose, cellulose, pectins and other components (Carpita and Gibeaut, 1993; Cassab and Varner, 1988; Tieman and Handa, 1994). As the ripening process begins, a series of biochemical changes occur at the cell wall resulting in the breakdown of cell wall polymers (Rose *et al*., 1997). The disintegration of cell walls can then lead to a reduction in intercellular adhesion, solubilization of pectins, depolymerization of hemicelluloses and loss of pectic galactose side chains.

Pectin is a main component of the primary cell wall, which plays a key role in maintaining the physical structure stability and mechanical strength of the cell wall. Galacturonic acid (GalA) is the major constituent of pectin, with a weight ratio of 70%. Pectin polysaccharides primarily comprise homogalacturonan (HG), xylogalacturonan (XGA), rhamnogalacturonan II (RG‐II) and rhamnogalacturonan I (RG‐I). HG is a linear polymer of the α‐1,4‐linked GalA, and XGA is a modified HG. In RG‐II, the HG backbone is complemented by lateral chains composed of 12 different sugars attached by 22 distinct linkages. The backbone of RG‐I is essentially a disaccharide repeating unit (α‐1,4‐D‐GalA‐α‐1,2‐L‐Rha) where the L‐rhamnose (Rha) can be branched with arabinan, galactan or arabinogalactan (Harholt *et al*., 2010). Because of the complex composition and spatial structure of pectic substances, many enzymes are required to catalyse the fruit softening process (Huber, 1984; Knee *et al*., 1977; Redgwell *et al*., 1997). Polygalacturonase (PG) and pectin methylesterase (PME) are the two most thoroughly characterized pectin‐modifying enzymes, but they are incapable of significantly affecting fruit texture (Grierson and Schuch, 1993; Hall *et al*., 1993). In tomato (*Solanum lycopersicum*), inhibition of PG activity to 1% level did not prevent pectin solubilization (Langley *et al*., 1994; Sheehy *et al*., 1988; Smith *et al*., 1990), and antisense inhibition of *PME* genes had relatively little effect on fruit softening (Tieman *et al*., 1992; Wen *et al*., 2013). Similar results were obtained in studies associated with endo‐glucanase (Brummell *et al*., 1999) and xyloglucan endotransglycosylase (De Silva *et al*., 1994). Additionally, suppressing other genes such as expansin and β‐galactosidase 4 had a moderate effect on regulating the softening process, but the changes were modest at most (Brummell *et al*., 1999, 2002; Smith *et al*., 2002).

Pectate lyases (PLs) (EC 4.2.2.2) are one kind of pectin‐modifying enzymes that are capable of cleaving glycosidic bonds through a β‐elimination mechanism between galacturonosyl residues (Marín‐Rodríguez *et al*., 2002; Willats *et al*., 2001). *PL‐like* sequences from higher plants were first reported in pollen (Wing *et al*., 1989). It was suggested that *PL* genes were required for the initial loosening of pollen cell walls to enable pollen tube growth and facilitate penetration of pollen (Taniguchi *et al*., 1995; Wu *et al*., 1996). *PL* sequences have been reported in climacteric fruit including banana (Domínguez ‐Puigjaner *et al*., 1997; Marín‐Rodríguez, 2001; Medina‐Suárez *et al*., 1997; Pilatzke‐Wunderlich and Nessler, 2001) and mango (Chourasia *et al*., 2006), as well as in nonclimacteric fruit including strawberry (Medina‐Escobar *et al*., 1997) and grape berries (Nunan *et al*., 2001). *PL* gene expression was first manipulated with transgenic approaches in strawberry, where the suppression of *PL* mRNA during ripening resulted in significantly firmer fruit (Jiménez‐Bermúdez *et al*., 2002; Santiago‐Doménech *et al*., 2008). Very recently, Uluisik *et al*. (2016) showed that silencing a *PL* gene in tomato led to targeted control of tomato softening, without affecting other aspects of ripening.

Tomato is one of the most important agricultural products in the world, as well as an important model for fleshy fruit ripening (Giovannoni, 2001). To further analyse the roles of pectate lyase in tomato, we identified 22 gene family members via *in silico* mining of the tomato genome (http://solgenomics.net/). One fruit softening‐specific gene Solyc03g111690 named *SlPL* in accordance with the recent study was cloned, and its functions were characterized through RNA interference (RNAi)‐mediated gene silencing. Silencing *SlPL* in tomato led to enhanced fruit firmness, extended fruit shelf‐life and reduced susceptibility to *Botrytis cinerea*. The findings indicated that *SlPL* could be a useful biotechnological tool in genetic engineering of fleshy fruit.

## Results

### Identification, sequence analysis and expression profiles of tomato *PL* genes

**Figure 1:**
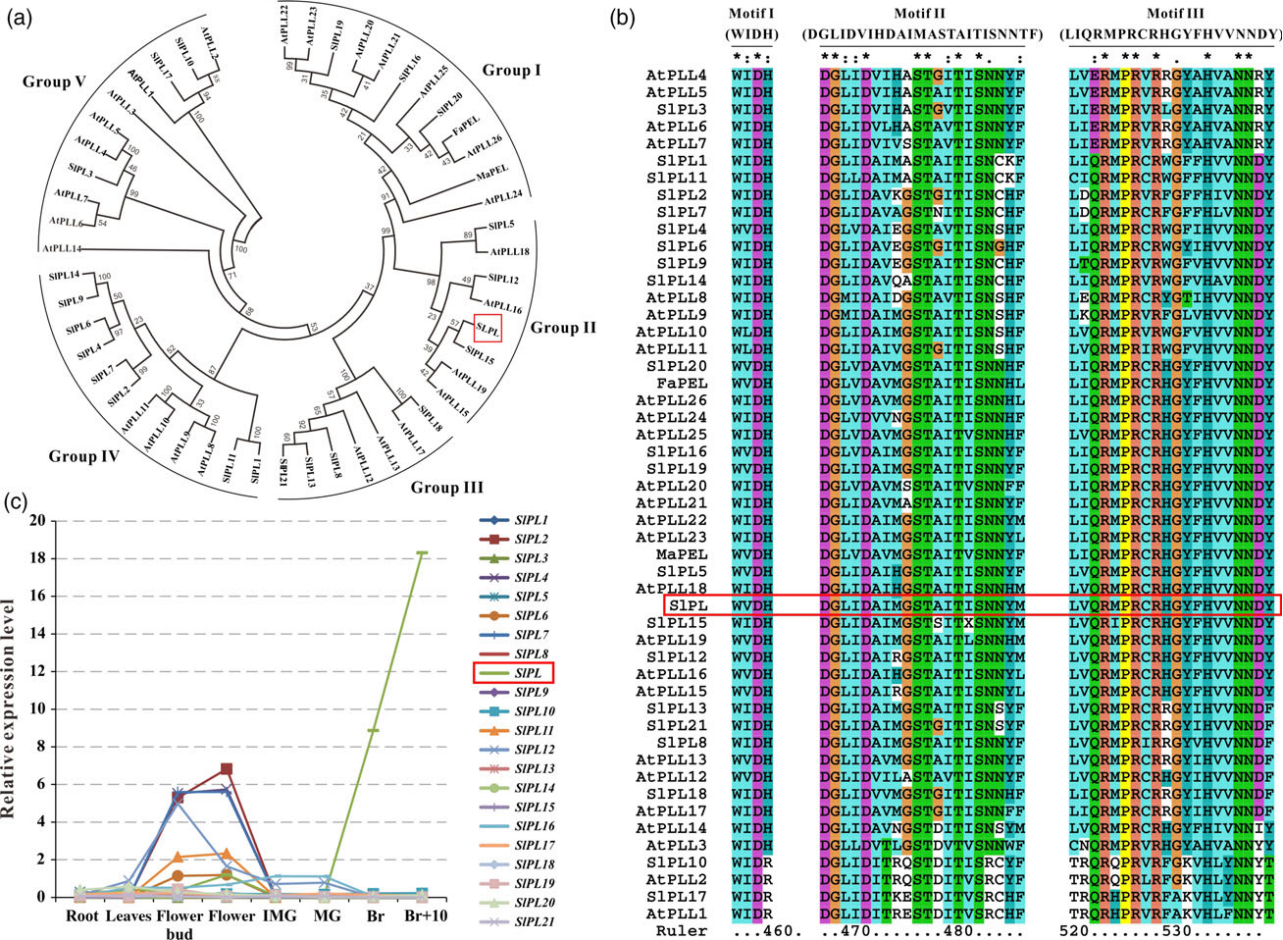
Phylogenetic analysis, multiple sequence alignment and expression patterns of pectate lyases (PLs). (a) Phylogenetic analysis of tomato PL gene family members, together with all 26 *Arabidopsis* PLs and two softening‐related PLs from strawberry (FaPEL) and banana (MaPEL), all of which were carried out by the neighbour‐joining method on MEGA6. (b) Three typical motifs (motif 1 WIDH, motif 2 DGLIDAIMASTAITISNNYF and motif 3 LIQRMPRCRHGYFHVVNNDY) of the PL amino acid sequences from tomato, *Arabidopsis*, strawberry and banana, comparatively analysed by Clustal X (Thompson *et al*., 2002). (c) Expression patterns of the tomato *PL* genes obtained from the TomExpress platform.

By mining the annotated tomato genome database (SOL Genomics Network, http://solgenomics.net/), we identified 22 tomato *PL* genes. A phylogenetic analysis of tomato PL sequences, together with all 26 *Arabidopsis thaliana* PLs and two softening‐related PLs from strawberry and banana (Data S1), was carried out using the neighbour‐joining method on MEGA6 to investigate the relationship between SlPLs and other PLs. The analysis revealed that the tomato genome carried all genes orthologous to those described previously in *Arabidopsis* and the tomato PL proteins could be divided into five subfamilies (Figure 1a), consistent with a previous investigation by Singh *et al*. (2011). Alignment of predicted PL domain sequences showed that three typical motifs, motif 1 (WIDH), motif 2 (DGLIDAIMASTAITISNNYF) and motif 3 (LIQRMPRCRHGYFHVVNNDY) could be recognized in the sequence (Figure 1b), thus further confirming the identity of the predicted *PLs* in tomato. Next, comprehensive transcriptomic profiling of 22 tomato *PLs* in vegetative and reproductive tissues was carried out using the online TomExpress platform and associated data‐mining tools (http://gbf.toulouse.inra.fr/tomexpress) (Figure 1c). Notably, we found that Solyc03 g111690 (hereafter designated as *SlPL*) had higher expression during fruit ripening and softening than other gene family members, suggesting that this gene might play important roles in regulating the fruit ripening/softening process.

### Detailed expression patterns of *SlPL* in wild‐type (WT) plants and hormone‐treated fruit

**Figure 2:**
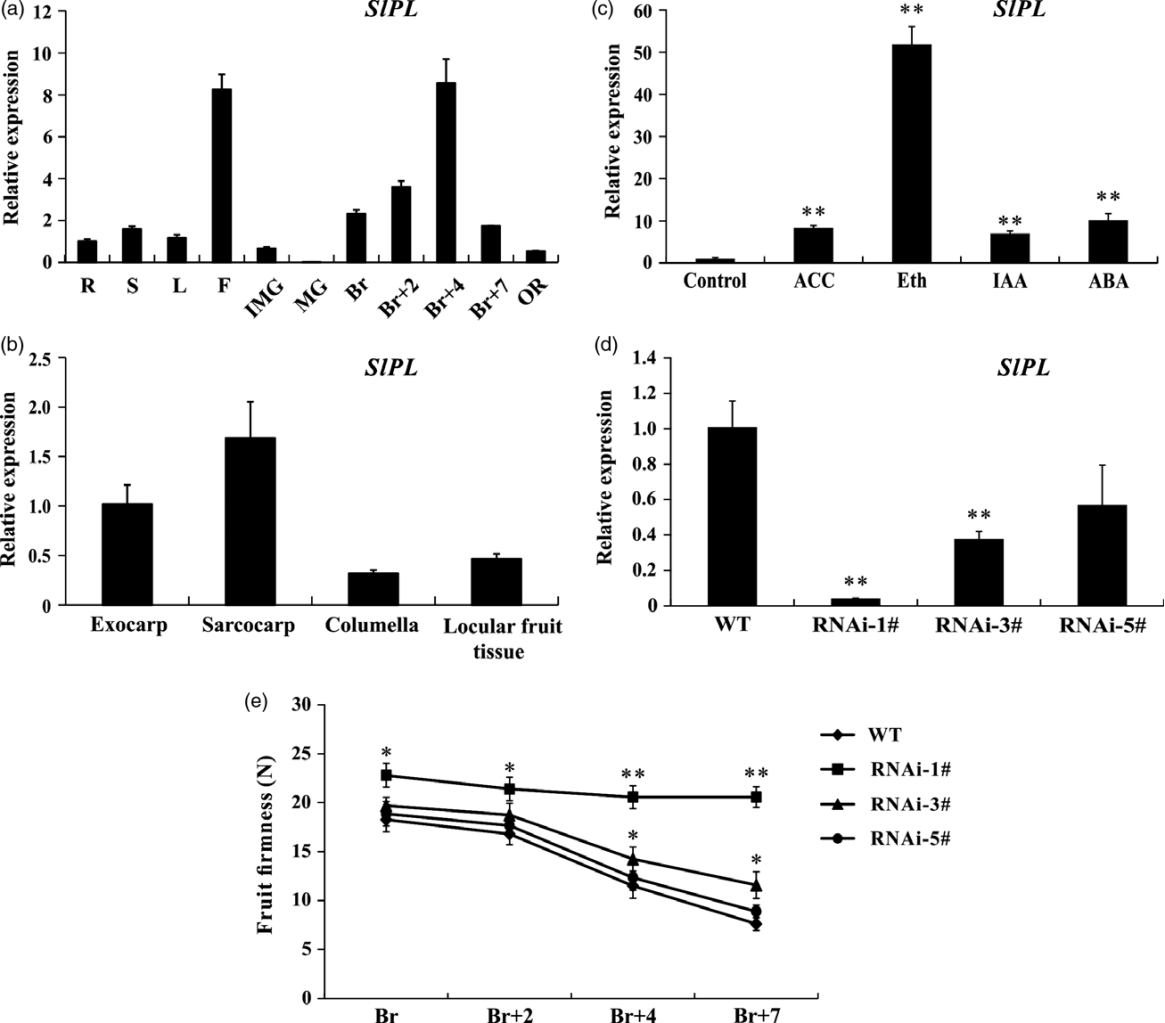
Expression patterns of *SlPL* (Solyc03g111690) and phenotype identification of *SlPL*‐RNAi transgenic tomato. (a) Expression of *SlPL* in various tissues including roots (R), stems (S), fully expanded leaves (L), flowers (F) and fruit at different developmental stages: immature green (IMG), mature green (MG), breaker (Br), 2 day after Br (Br+2), 4 day after Br (Br+4), 7 day after Br (Br+7, RR) and over ripe (OR). (b) Spatial expression of *SlPL* in WT fruit at Br+4 stage. (c) Response of *SlPL* to several ripening‐related hormones after treatment for 96 h. ACC, 1‐aminocyclopropane‐1‐carboxylic acid; Eth, ethephon; IAA, indole‐3‐acetic acid; and ABA, abscisic acid. (d) *SlPL* mRNA level in fruit of three independent RNAi lines (RNAi‐1#, RNAi‐3# and RNAi‐5#). (e) Phenotype of enhanced fruit firmness at different fruit development stages in RNAi lines. Quantitative PCR data represent mean values for three independent biological replicates (*n* = 3). The fruit firmness values represent the means ± standard error (SE) of 20 fruit per line at each stage. * and ** represent significant differences between *SlPL*‐RNAi lines and WT by *t*‐test with *P* < 0.05 and *P* < 0.01, respectively.

To assess the potential roles of *SlPL* throughout tomato development, we conducted detailed quantitative real‐time PCR (qRT‐PCR) to examine its transcription in different tissues. The expression levels were lower in roots (R), stems (S) and leaves (L) but relatively high in flowers (F). During tomato fruit development, expression of *SlPL* was relatively low in immature green (IMG) fruit and was negligible in mature green (MG) fruit. However, the expression level was drastically enhanced from the breaker stage (Br) and reached a maximum at 4 d after Br (Br+4) (Figure 2a). The observations were basically consistent with the data obtained from the TomExpress platform, further implying a possible crucial role of *SlPL* during fruit ripening/softening. Therefore, we isolated the total RNA from exocarp, sarcocarp, columella and locular tissue from fruit at Br+4 stage for qPCR analysis. The results showed that the *SlPL* expression level was relatively high in exocarp and sarcocarp (Figure 2b), indicating that *SlPL* functioned mainly in pericarp. To clarify the regulation of ripening‐related phytohormones on softening‐related genes, we tested the expression of *SlPL* in MG fruit of wild type (WT), respectively, treated with 1‐aminocyclopropane‐1‐carboxylic acid (ACC), ethephon (Eth), indole‐3‐acetic acid (IAA) and abscisic acid (ABA). The mRNA levels were significantly up‐regulated especially after the Eth treatment (Figure 2c). The results demonstrated that different types of phytohormones affected not only ripening but softening as well.

### Down‐regulation of *SlPL* enhances fruit firmness

To further elucidate the function of *SlPL*, an RNAi construct targeting the fragment of this gene was created and transformed into WT tomato cv. Micro‐Tom plants via *Agrobacterium tumefaciens* mediated T‐DNA transfer. Real‐time PCR results showed that *SlPL* transcripts were significantly decreased in the transgenic lines compared with WT (Figure 2d). We next measured the fruit firmness at four different ripening stages: breaker (Br), 2 day after breaker (Br+2), Br+4 and 7 day after breaker (Br+7) (Figure 2e). The firmness in WT fruit decreased gradually with fruit ripening, with values of 18.3, 16.8, 11.5 and 7.6 newton (N) for each stage, respectively. In *SlPL‐*RNAi lines, it seems that the fruit firmness variation was deeply associated with the *SlPL* transcripts because no difference was observed for RNAi‐5# compared with WT, which had a transcriptional inhibition rate of 40%. For RNAi‐3#, with transcriptional inhibition rate of about 60%, the firmness was moderately enhanced. For RNAi‐1#, with nearly 95% inhibition of *SlPL*, the values of fruit firmness of 22.8, 21.4, 20.6 and 20.4 N for Br, Br+2, Br+4 and Br+7, respectively, showed minimum reductions in firmness throughout the experiment.

### Silencing of *SlPL* leads to compact cells in fruit pericarp

**Figure 3:**
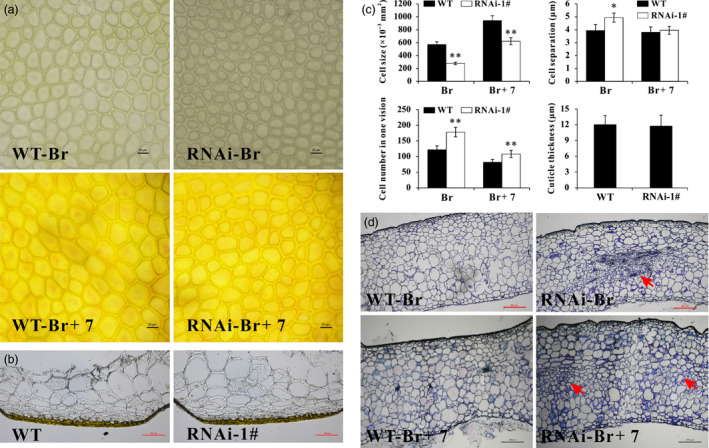
Differences of fruit pericarp cells in *SlPL*‐RNAi lines and WT. (a) Epidermal cells at the inner surface at Br and Br+7 stages from identical positions of different fruit. (b) Micrographs of unstained fruit epicarp sections from ripe tomatoes of WT and *SlPL*‐RNAi lines for the visualization of the cuticle. Bars = 100 μm. (c) Statistical analysis regarding epidermal cell size, cell number, cell separation and cuticle thickness of *SlPL*‐RNAi and WT fruit. Data are shown as means ± SE. * and ** indicate significant differences between *SlPL*‐RNAi lines and WT with *P* < 0.05 and *P* < 0.01, respectively, as determined by *t*‐test. (d) Paraffin transverse sections of pericarp tissues at Br and Br+7 stage fruit stained with toluidine blue. The differences in mesocarp tissues in *SlPL*‐RNAi fruit were indicated by red arrows. Sections were isolated from the same location in all fruit.

As the pericarp is important in determining the rate of expansion and mechanical support for the whole fruit (Bargel and Neinhuis, 2005), histological analysis of the inner surface of the epidermis was conducted to compare the cell walls in WT and transgenic fruit. Increased cell separation was observed in *SlPL*‐RNAi fruit compared with WT (Figure 3a), and the corresponding statistical data were shown in Figure 3c. Also, the epidermal cells were smaller in *SlPL*‐RNAi transgenic fruit, leading to a larger number of epidermal cells (Figure 3a and c). Although cuticle is synthesized by the epidermal cell layer of the fruit pericarp, histological sectioning of sectors from fruit epicarp showed no difference in cuticle structure and thickness between WT and *SlPL*‐RNAi lines (Figure 3b and c). However, cell sizes and number as well as cell layer patterning of the whole pericarp changed. Consistent with the transverse sections, photographs revealed compact cells and increased cell numbers in exocarp, mesocarp and endocarp of RNAi fruit compared with WT (Figure 3d). Intriguingly, the phenomenon of ‘compact cells’ was reinforced in mesocarp, indicating that the site of action for SlPL was mostly in mesocarp cells rather than other cell types. These results suggest that the *SlPL* gene was involved in pericarp cell wall rearrangement during fruit softening.

### The *SlPL*‐RNAi fruit exhibits longer shelf‐life

**Figure 4:**
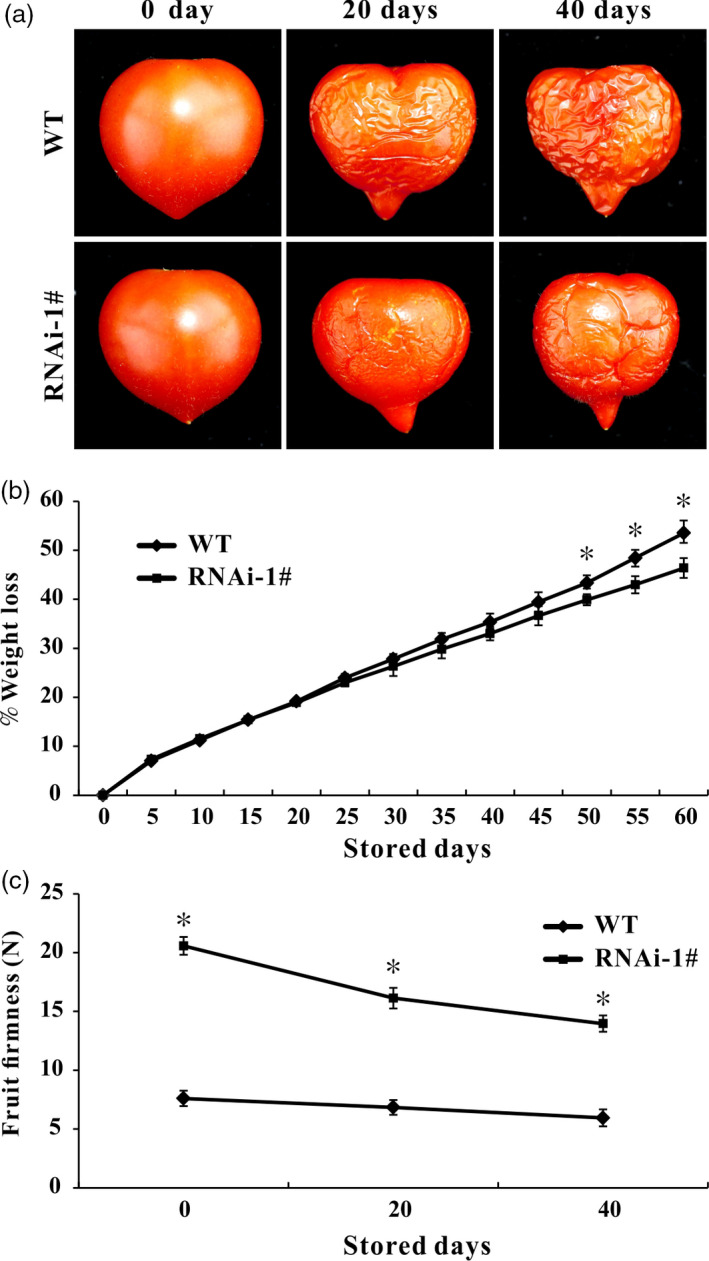
Suppression of *SlPL* extended fruit shelf‐life. (a) Red ripe (RR) fruit from *SlPL*‐RNAi lines and WT after storage at room temperature (23–25 °C and 55%–60% relative humidity) for 0, 20 and 40 day. (b) Physiological loss of water (PLW) in *SlPL*‐RNAi and WT fruit. The weight loss per fruit was calculated every 5 day during 0–60 day after storage. Values represent means ± SE (*n* = 20). * indicates significant differences between *SlPL*‐RNAi and WT fruit with *P* < 0.05, as determined by *t*‐test. (c) Change of fruit firmness in RNAi lines and WT. The fruit firmness per fruit was measured after storage of 0, 20 and 40 day. Average values were calculated for 20 individual fruit, and standard error of mean (SEM) was marked out. * indicates significant differences between *SlPL*‐RNAi and WT fruit with *P* < 0.05, as determined by *t*‐test.

The WT and *SlPL*‐RNAi fruit were harvested at Br+7 and stored at 23–25 °C with 55%–60% relative humidity until they reached complete deterioration. WT fruit were wrinkled after 6–7 day of storage, whereas *SlPL*‐RNAi fruit showed similar symptoms of senescence after 10–15 day of storage. At the time that effusion of juice and loss of texture and integrity occurred in WT fruit, the texture and fruit firmness were retained in *SlPL*‐RNAi fruit. Comparative analysis of images of fruit after storage of 0, 20 and 40 day showed less wrinkles for *SlPL*‐RNAi than WT fruit (Figure 4a). *SlPL*‐RNAi fruit exhibited lower physiological loss of water (PLW) than WT after 20 day of storage, although PLW did not differ in the first 20 day of storage (Figure 4b). During storage, fruit firmness was much higher in *SlPL*‐RNAi than in WT (Figure 4c).

### *SlPL*‐RNAi fruit with reduced susceptibility to *B. cinerea*

**Figure 5:**
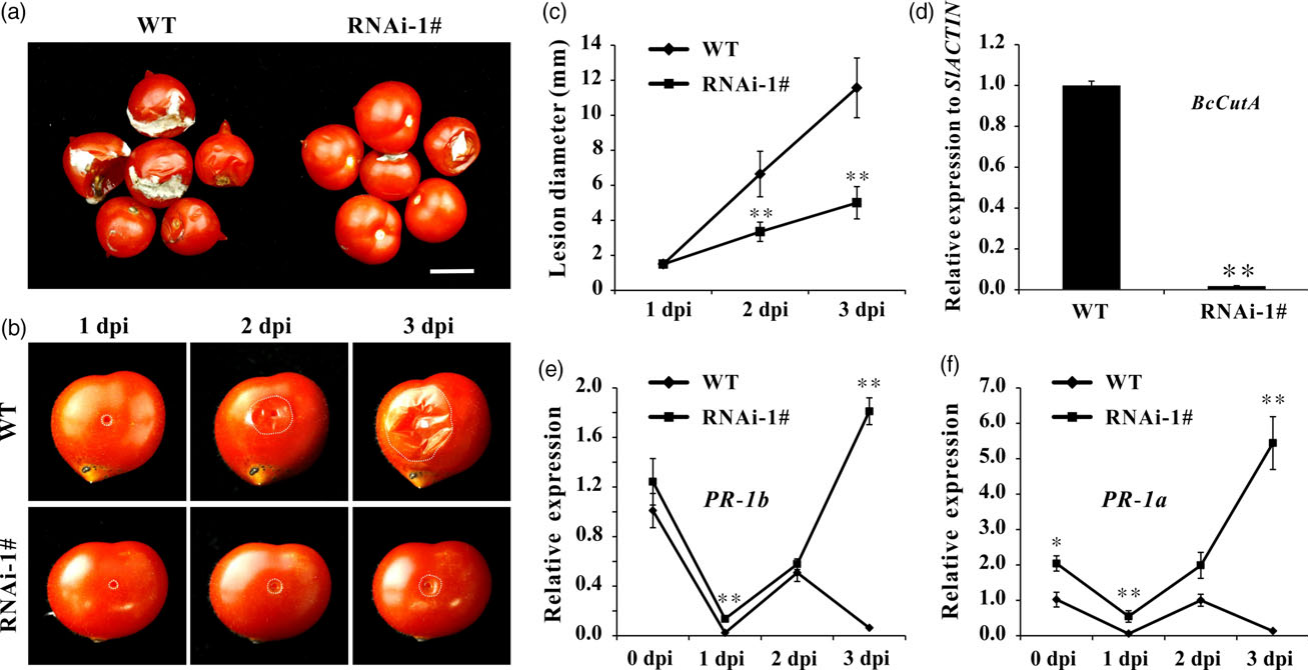
Suppression of *SlPL* in tomato decreased pathogen susceptibility. (a) Symptoms of intact WT and *SlPL*‐RNAi fruit after spraying of spores of *B. cinerea* (B05.10). Compared to rapid softening and collapsing symptoms in WT, fruit of the *SlPL* silenced line showed little mycelium at 5 days postinoculation (dpi). The scale bar represents 1 cm. (b) Symptoms of wounded WT and *SlPL*‐RNAi fruit after inoculation with *B. cinerea* (B05.10) at 1, 2 and 3 dpi. White dots represent the lesion margins. (c) Lesion diameter was measured at 1, 2 and 3 dpi. Error bars show the SE of means (*n* = 6). ** refers to significant differences between RNAi‐1# and WT with *P* < 0.01 determined by t‐test. (d) Quantitative PCR revealed more *B. cinerea* growing on the WT than on *SlPL*‐RNAi fruit at 3 dpi. The *B. cinerea* growth was calculated by comparing the ratio of *B. cinerea* DNA to tomato DNA. (e) Relative expression level of *PR‐1b* in RNAi‐1# and WT fruit (Br+14) after inoculation with *B. cinerea* at 0, 1, 2 and 3 dpi. (f) Relative expression level of *PR‐1a* in RNAi‐1# and WT fruit (Br+14) after inoculation with *B. cinerea* at 0, 1, 2 and 3 dpi. For qPCR analysis, the data represent mean values for three independent biological replicates. * and ** indicate significant differences between *SlPL*‐RNAi lines and WT with *P* < 0.05 and *P* < 0.01, respectively, as determined by *t*‐test.

*Botrytis cinerea* (B05.10) is the causal agent of grey mould disease, which is one of the most important postharvest pathogens of tomatoes (Williamson *et al*., 2007). When intact tomato fruit was sprayed with *B. cinerea* spore suspension, the WT fruit showed severe symptoms of infection with visible mycelium at 5 day postinoculation (dpi). However, the extent of infection was less severe in *SlPL*‐RNAi fruit (Figure 5a). When wounded fruit were inoculated with *B. cinerea* spore suspension, the size of the lesions did not increase at 1 dpi, indicating that establishment of the fungus needed about 24 h after inoculation. From 2 dpi, the spread of the infected area was greater in WT fruit. At 3 dpi, the average size of lesions in *SlPL*‐RNAi fruit was significantly less than that in WT, indicating reduced susceptibility to *B. cinerea* infection (Figure 5b). The lesion diameters at 1, 2 and 3 dpi in WT were nearly twice that in *SlPL*‐RNAi fruit (Figure 5c). The qPCR confirmed significantly more *B. cinerea* growing on WT fruits (Figure 5d). After invasion by *B. cinerea*, the expression of pathogen‐related genes (*PR‐1b* and *PR‐1a*) were down‐regulated in WT, while in RNAi fruit, their expressions were initially suppressed but significantly increased at 3 dpi. Notably, the mRNA levels were always higher in RNAi than WT fruit at all times after infection (Figure 5e and f).

### Altered cell wall components and enhanced antioxidant capacity by silencing *SlPL*

**Figure 6:**
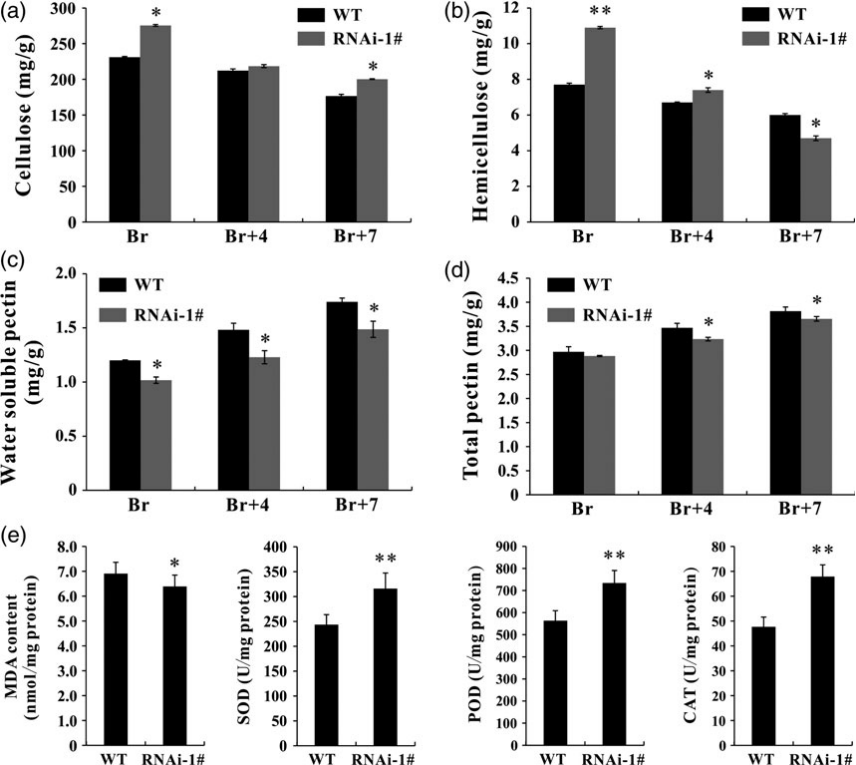
Pericarp cell wall components and antioxidant capacity in *SlPL*‐RNAi and WT fruit. (a) Content of cellulose (mg/g) in *SlPL*‐RNAi and WT fruit at Br, Br+4 and Br+7 stages. (b) Content of hemicellulose (mg/g) in *SlPL*‐RNAi and WT fruit at Br, Br+4 and Br+7 stages. (c) Content of water‐soluble pectin (WSP) (mg/g) in *SlPL*‐RNAi and WT fruit at Br, Br+4 and Br+7 stages. (d) Content of total pectin (mg/g) in *SlPL*‐RNAi and WT fruit at Br, Br+4 and Br+7 stages. (e) The malondialdehyde (MDA) level (nmol/mg protein) and antioxidant enzyme activity of superoxide dismutase (SOD), peroxidase (POD) and catalase (CAT) (U/mg protein) in *SlPL*‐RNAi and WT fruit at Br+14 stage. The error bars represent the SE calculated from three measurements for each cell wall material. * and ** indicate significant differences between *SlPL*‐RNAi and WT plants with *P* < 0.05 and *P* < 0.01, respectively, as determined by *t*‐test.

The main components of plant cell walls are cellulose and pectin. In this study, contents of cellulose, hemicellulose, total pectin and water‐soluble pectin in WT and *SlPL*‐RNAi fruit were measured to determine whether the inhibition of *SlPL* led to changes in cell wall material. Three ripening stages were chosen for data collection: Br, Br+4 and Br+7. At each stage, the cellulose content in *SlPL*‐RNAi fruit (275.5, 218.5 and 200.3 mg/g, respectively) was higher than that in WT (231.1, 212.2 and 176.6 mg/g, respectively) (Figure 6a). The hemicellulose content in *SlPL*‐RNAi fruit (10.851 and 7.444 mg/g, respectively) was also greater than in WT (7.708 and 6.656 mg/g, respectively) at Br and Br+4 stages (Figure 6b). In contrast, the water‐soluble pectin was significantly reduced in *SlPL*‐RNAi fruit (Figure 6c), suggesting that most pectin in *SlPL*‐RNAi fruit cell walls was covalently attached to the wall matrix, and that down‐regulation of *SlPL* inhibited pectin degradation. Due to the decrease of water‐soluble pectin, the *SlPL*‐RNAi fruit also showed a slight reduction in total pectin (Figure 6d).

Malondialdehyde (MDA) is a by‐product of lipid peroxidation and can be used to evaluate the damage by oxidative stress during tissue senescence. Superoxide dismutase (SOD), peroxidase (POD) and catalase (CAT) are three important antioxidant enzymes. MDA level, together with SOD, POD and CAT activities, was monitored in fresh red ripe (RR) fruit. Compared with WT, there were lower MDA levels and higher SOD, POD and CAT enzyme activities in *SlPL*‐RNAi fruit (Figure 6e), suggesting that it had stronger antioxidant ability.

### Differentially expressed genes (DEGs) based on RNA sequencing data

**Figure 7:**
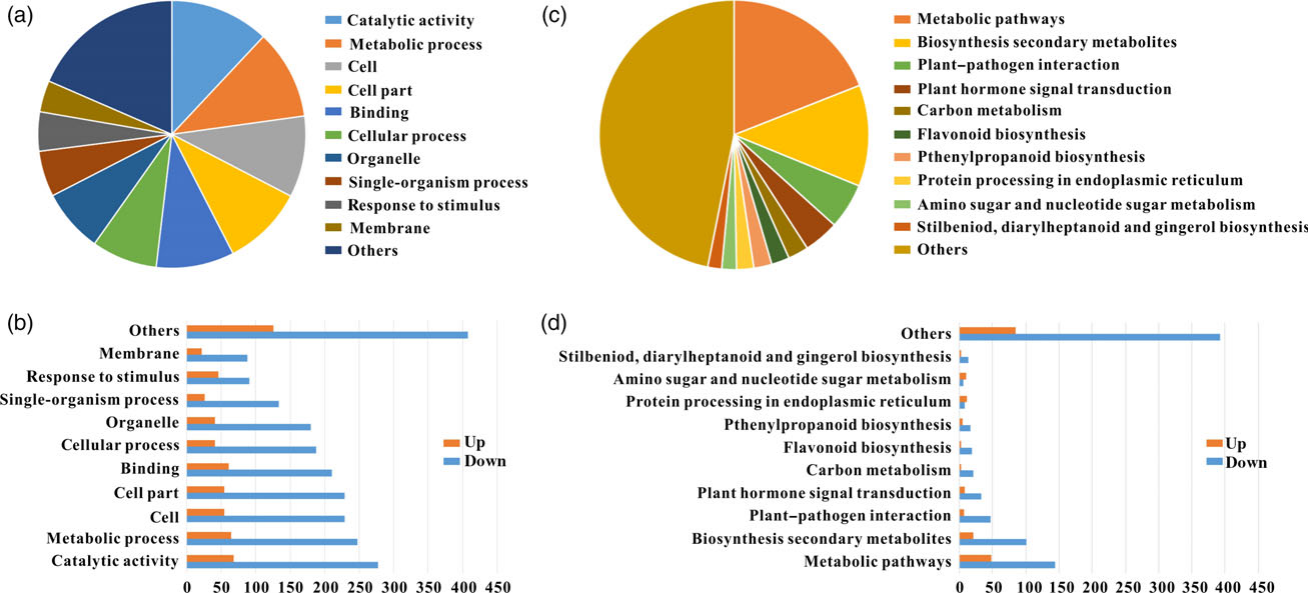
Classification of DEGs with RNA‐Seq data. (a) GO classification and functional annotation of the 943 genes showing more than twofold differences in expression between RNA‐1# and WT fruit at Br+4 stage. (b) Functional classification of all differentially expressed genes at Br+4 stage with GO classification. (c) Pathway and KEGG classification and functional annotation of the 943 genes showing more than twofold differences in expression between RNA‐1# and WT fruit at Br+4 stage. (d) Functional classification of all differentially expressed genes at Br+4 stage with pathway and KEGG classification.

Gene expression profiles of WT and *SlPL*‐RNAi fruit were compared using RNA sequencing (RNA‐Seq) data at Br+4 stage. A twofold difference in expression level was set as the threshold for data significance. In RNAi fruit, 943 genes consisting of 181 up‐regulated and 762 down‐regulated genes showed apparent differential expression. The DEGs were functionally identified using gene ontology (GO) analysis and categorized into 11 main categories (Figure 7a and b). In addition, using Kyoto Encyclopedia of Genes and Genomes (KEGG) pathway analysis, many DEGs were found to be involved in cell wall metabolism and plant–pathogen interaction (Figure 7c and d). Exhaustive analysis showed that 49 DEGs were phytohormone related, including 22 with ethylene, 14 with auxin, four with gibberellins, five with abscisic acid, two with jasmonate acid, one with salicylic acid and one with cytokinins (Table S2). Transcription factors (TFs) are an important part of functional genomics and participate in various regulatory networks in higher plants. We searched the RNA‐Seq data and found 68 DEGs encoding TFs. The dominant TF families were the ERF (16 DEGs), bHLH (7 DEGs), MADS‐box (7 DEGs), WRKY (7 DEGs), AP2 (5 DEGs), bZIP (4 DEGs), B3 (4 DEGs), MYB (3 DEGs), NAC (2 DEGs), C2H2 (2 DEGs) and GRAS (1 DEG) (Table S3), suggesting important roles of these TFs in fruit ripening/softening. Moreover, functional annotation revealed that many DEGs were involved in cell wall modification (63 DEGs), oxidative stress (39 DEGs) and pathogen resistance (78 DEGs) (Tables S4 and S5). The transcriptomic data partly explained the molecular basis underlying the extended shelf‐life and pathogen resistance of the *SlPL*‐RNAi fruit.

**Figure 8:**
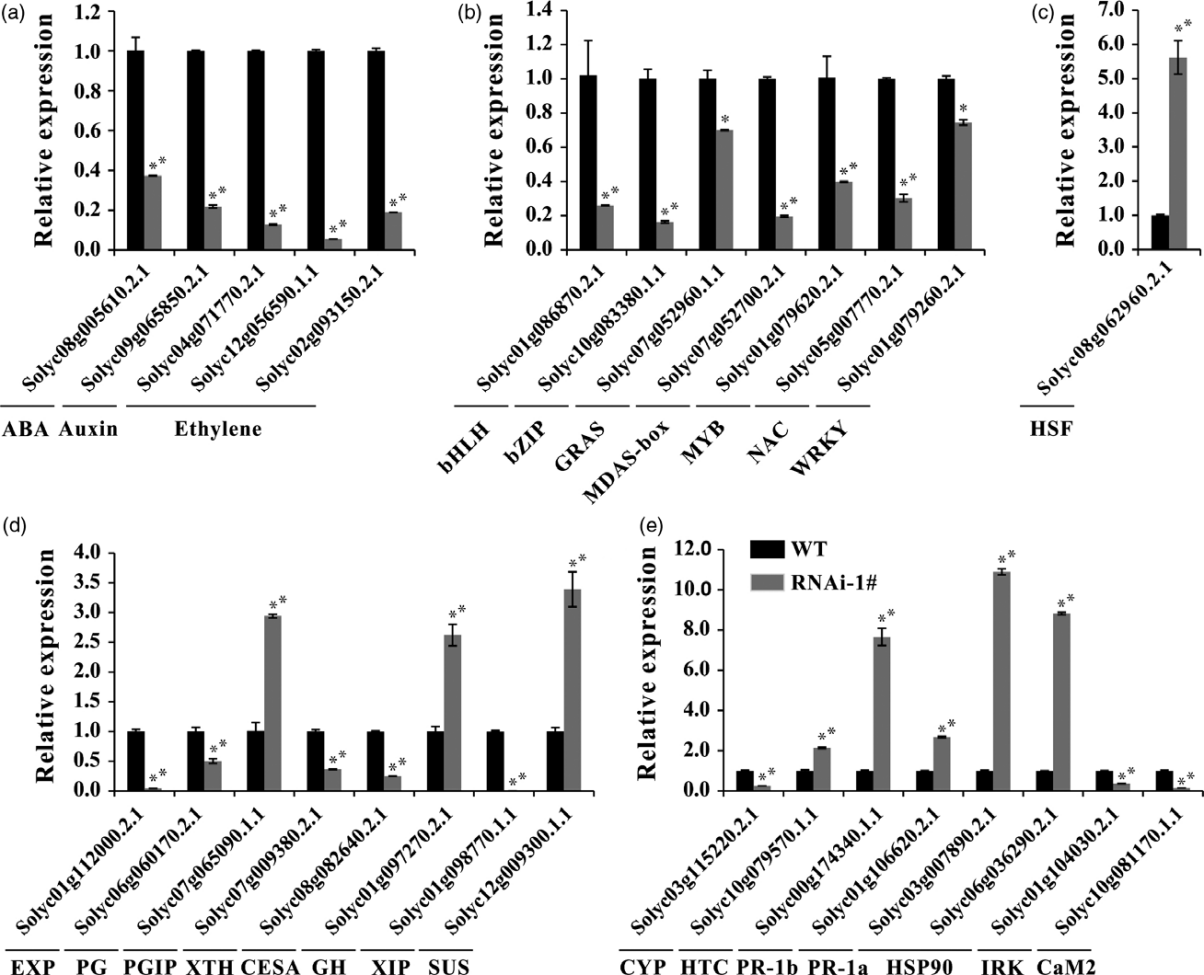
Verification of selected DEGs by real‐time PCR. (a) Relative expression of genes related to phytohormones ABA, auxin and ethylene. (b) and (c) Relative expression of genes related to transcription factors. (d) Relative expression of genes related to cell wall modification and degradation. (e) Relative expression of genes related to oxidative stress and pathogen resistance. Error bars show the SE between three biological replicates performed (*n* = 3). * and ** indicate *P* < 0.05 and *P* < 0.01, respectively.

To validate our transcriptomic data, 29 genes were chosen for supplementary qRT‐PCR analysis in WT and RNAi fruits (Figure 8). The selected genes comprised five phytohormone related, eight TF, eight cell wall‐processing and eight stress‐related genes. The qRT‐PCR results were highly consistent with transcriptomic data for all tested genes.

## Discussion

Fruit softening is a result of degradation/modification of cell wall polymers by the action of cell wall associated hydrolases (Brummell and Harpster, 2001; Fisher and Bennet, 1991). However, many enzymes cannot be exploited to genetically control the softening of fruit. In the past few decades, pectate lyase has been documented as a potential candidate for manipulating fruit softening in several fruit crops, including strawberry, banana, mango and grape (Chourasia *et al*., 2006; Marín‐Rodríguez *et al*., 2002; Payasi and Sanwal, 2003). Very recently, Uluisik *et al*. (2016) revealed that suppressing a *PL* gene in tomato could enhance fruit firmness and extend shelf‐life without impacting the flavour, aroma, taste and nutritional value. In the present study, we identified 22 members of the *PL* gene family in tomato and divided them into five subfamilies (Figure 1a). Using a RNAi strategy, we further characterized a ripening/softening‐specific gene *SlPL* (Solyc03 g111690). Due to the high homology of sequences (over 80%) between transgenic construction and several other *PL* genes expressed in immature fruit (*SlPL16*,* SlPL5* and *SlPL19*), qPCR analysis was performed at different development stages: IMG, MG and Br+4. The results demonstrated that these genes were affected to some extent at one or two stages, while *SlPL* was severely inhibited at all stages especially during fruit ripening (Figure S1). The expression of other two genes *SlPL12* (Solyc05 g014000) and *SlPL15* (Solyc06 g071840) which were closed to *SlPL* in phylogenetic tree also showed no significant suppression at RR fruit (Figure S1e). These findings indicated that *SlPL* was most relevant to the above phenotype while other gene family members might have made minor contributions. In agreement with the work by Uluisik *et al*. (2016), the *SlPL*‐RNAi lines were capable of producing fruit with enhanced firmness and extended shelf‐life (Figures 2 and 4) without negative effects on vegetative growth, fruit development, days to maturity, and fruit yield except for a decreased weight of seeds and ratio of seed germination (Table S6).

Fruit softening is usually accompanied by a reduction in cell turgor, due to increasing concentrations of solutes in the cell wall space and wall loosening (Shackel *et al*., 1991). A tomato mutant cultivar ‘delayed fruit deterioration’ (DFD) showed minimal transpirational water loss and substantially elevated cellular turgor likely due to specific compositional or ultrastructural characteristics of the fruit cuticle (Saladié *et al*., 2007). Although the cuticle can function as an important barrier to reduce water loss and extend shelf‐life (Segado *et al*., 2016), it showed very similar structure and thickness between WT and RNAi fruit in our research (Figure 3b and c). This result is in accordance with the report by Uluisik *et al*. (2016) in which the *SlPL*‐RNAi fruit showed longer shelf‐life. In their study, the cuticle waxes of *SlPL*‐RNAi and WT fruit did not significantly differ, although genes involved in epidermal regulation and cuticle development such as *PROTODERMAL FACTOR 2‐like* (Solyc06g035940) and *CER1* (Solyc03g065250) were up‐regulated (Uluisik *et al*., 2016). Additionally, Giménez *et al*. (2015) demonstrated that greater thickness of the cuticle may not affect the breaking stress. Generally, an increase in cell size is indicative of a positive turgor pressure that facilitates cell expansion. However, anatomical observations showed that the size of epidermal cells was reduced in RNAi fruit (Figure 3), suggesting that the enhanced fruit firmness in *SlPL*‐RNAi transgenic lines was not caused by cell expansion. The main effect of SlPL is in the tricellular junction zones of pericarp parenchyma cells, and it breaks down the cross‐linked homogalacturonan (HG) polymers in both middle lamella and tricellular junctions (Uluisik *et al*., 2016). Consistently, we found more compact parenchyma cells in RNAi fruit pericarps than in WT (Figure 3d). In addition, silencing *SlPL* would inhibit the eliminative cleavage of de‐esterified pectin. Compared with WT, contents of cellulose and hemicelluloses were significantly higher in RNAi fruit, while the water‐soluble pectin and total pectin were lower (Figure 6).

*Botrytis cinerea* is a necrotrophic fungal pathogen capable of killing plant cells and proliferating on dead tissues (Prins *et al*., 2000). Simultaneous suppression of *LePG* and *LeExp1* in ripening tomato fruit reduced wall disassembly, slowed fruit softening and decreased ripening‐associated susceptibility to *B. cinerea* (Cantu *et al*., 2008). Unfortunately, suppression of *LePG* or *LeExp1* alone did not reduce the susceptibility of fruit to *B. cinerea* infection, suggesting that PG and Exp may act cooperatively against *B. cinerea* invasion in tomato (Brummell *et al*., 1999, 2002; Kalamaki *et al*., 2003; Powell *et al*., 2003). It is remarkable that the *SlPL*‐RNAi fruit in our study showed reduced susceptibility to *B. cinerea* (Figure 5). These findings support the ability of *SlPL* in specific control of fruit softening and *B. cinerea* invasion. As the expression of *SlPL5* was also up‐regulated during fruit ripening in WT and co‐suppressed in *PL*‐RNAi fruit (Figure S1), we monitored expression of *SlPL* and *SlPL5* after *B. cinerea* infection, the trend of *SlPL* expression in RNAi fruit after inoculation was similar to that in WT. However, the mRNA level of *SlPL5* showed no significant change at 2 or 3 dpi in RNAi fruit after *B. cinerea* treatment (Figure S2), demonstrating that *SlPL* may have contributed to the observed pathogen resistance rather than *SlPL5*. Actually, pectate lyase can activate defence systems by releasing plant cell wall oligogalacturonides, which can then function as defence elicitors (De Lorenzo *et al*., 1991). *Del*/*Ros1* tomato fruit showed decreased *B. cinerea* susceptibility due to higher anthocyanin accumulation, which contributed to attenuated oxidative damage (Zhang *et al*., 2013). SOD, POD and CAT are important antioxidant enzymes serving as efficient scavengers of reactive oxygen species to avoid oxidative damage. The *SlPL*‐RNAi RR fruit possessed lower MDA content and higher antioxidant enzyme activities compared with WT fruit (Figure 6e).

In our RNA‐Seq data, 943 genes showed more than twofold difference in expression level at Br+4 stage compared with WT. This result differed from the recent study, which showed fewer than 120 genes were altered in transcriptomes at the orange stage (Uluisik *et al*., 2016). This difference may be because Uluisik *et al*. used a different cultivar. Additionally, our results showed suppression of *SlPL5* and *SlPL19* (Figure S1). The latter was highly expressed in immature fruit of WT. These gene expression changes could be also responsible for the differences between our findings and those reported by Uluisik *et al*. (2016) with respect to cell size and differential gene expression. The DEGs were associated with plant hormones, fruit ripening, cell wall degradation, as well as pathogens. Ethylene, IAA and ABA are important hormones related to fruit ripening, to which the softening‐related gene *SlPL* can respond (Figure 2c). Moreover, the hormone‐related DEGs were also found in *SlPL*‐RNAi fruit (Figure 8). Genes encoding polygalacturonase (*SlPG*) and xyloglucan endotransglycosylase (*SlXTH*), and other genes involved in cell wall softening, showed substantially lower expression in *SlPL*‐RNAi fruit during ripening (Figure 8). Although the silencing of individual genes might only have minor effects on softening, the simultaneous suppression of a number of cell wall modification enzymes would likely result in significant reductions in the rate of fruit softening (Giovannoni, 2004; Goulao and Oliveira, 2008; Powell *et al*., 2003).

In conclusion, we demonstrated that SlPL was undeniably an important pectinase related to cell wall disassembly and fruit softening in tomato. The extended shelf‐life and disease resistance of *SlPL*‐RNAi fruit revealed the great value of pectate lyase.

## Materials and methods

### Plant materials and growth conditions

*Solanum lycopersicum* cv. Micro‐Tom was selected as the wild type (WT). Tomato plants were grown in a greenhouse with standard conditions (16/8 h and 25 °C/18 °C of day/night, 80% humidity and 250 μmol/m^2^/s light intensity) and watered daily. The tissues of roots (R); stems (S); leaves (L); flowers (F); IMG fruit; MG fruit; Br stage fruit; yellow fruit, Br+2; orange fruit, Br+4; RR fruit, Br+7; Br+14; and over ripe fruit (OR) were collected from WT and transgenic tomato plants. The tissues of exocarp, sarcocarp, columella and locular fruit tissues of WT Br+4 stage fruit were also collected. For RNA extraction and cell wall composition analysis, samples were collected, frozen in liquid nitrogen immediately and stored at –80 °C. For cytological, texture and postharvest biology analyses, experiments were performed using fresh fruit material.

### Tomato *PL* gene family analysis and expression data mining

In the SOL Genomics Network (http://solgenomics.net), ‘pectate lyase’ was inputted in the annotation text to search unigene families. Multiple sequences alignments of the amino acid sequences of PL proteins from different species were analysed by ClustalX 1.83. A phylogenetic tree was constructed using MEGA6 software, and statistical confidence of the nodes of the tree was calculated using 10 000 bootstrap replicates. Expression patterns of 22 tomato *PL* genes during vegetative and reproductive development were predicted with TomExpress bioinformatics platform (http://gbf.toulouse.inra.fr/tomexpress).

### Vector construction and plant transformation

For RNAi vector construction, 377‐basepairs DNA sequences of *SlPL* were selected, and the sense and antisense orientation fragments were then amplified with primers listed in Table S1. Then, the amplified products were digested with EcoR1/Kpn1 and XbaI/BamH1, respectively, and linked into the pHANNIBAL vector (Wesley *et al*., 2001). Finally, the double‐stranded RNA expression unit, cauliflower mosaic virus 35S promoter and OCS terminator were inserted into the plant binary vector pBIN19 with *Sac*I and *Xba*I restriction sites. The *SlPL*‐RNAi binary plasmid was transferred into *Agrobacterium* strain GV3101, and *Agrobacterium*‐mediated transformation was performed following the protocols described by Fillatti *et al*. (1987). Transgenic plants were identified by PCR with primers of neomycin phosphotransferase II (NPTII) (Table S1). The positive transgenic plants were selected and used for subsequent experiments.

### RNA isolation and qPCR analysis

Total RNAs were extracted using Trizol reagent following the protocol provided by the manufacturer (Invitrogen) and treated with *DNase* I (Thermo Scientific). About 2 μg of total RNA from each sample was used for first‐strand cDNA synthesis. For qPCR, the reaction was performed using SyBR Green PCR Master Mix (CWBIO, China). The procedures of PCR amplification consisted of an initial incubation at 95 °C for 20 s, followed by 40 cycles of 95 °C for 3 s and 60 °C for 30 s. PCR products were monitored using Bio‐Rad CFX connect (Bio‐Rad). Each sample was amplified in triplicate, and normalization was performed by comparative CT method. *SlActin* (Solyc03g078400) was selected as the internal reference. All primers for qPCR are listed in Table S1.

### Treatments of tomato WT MG fruit with different hormones

For hormone treatment experiments, tomato fruit at MG stage was used. Final concentration of 100 μm ACC, IAA, ABA and 0.4% ethephon in solution (pH 5.6) containing 10 mm MES and 3% sorbitol (all Sigma‐Aldrich products) was prepared in the above order, and buffer injection was performed according to a preliminary study (Orzaez *et al*., 2006). After treatment, fruit were incubated in a culture room at 26 °C, under 16/8‐h light/dark cycle with a light intensity of 100 μmol/m^2^/s. Pericarp was collected after 96 h and frozen at –80 °C for further analysis.

### Fruit texture analysis

Fruit firmness was determined based on compression mass and skin puncture strength of fresh intact fruit collected at different fruit ripening stage, including MG, Br, Br+2, Br+4 and Br+7 stages, using a GY‐4 digital fruit sclerometer (Aiwoshi, China). The analyser was equipped with a circular probe of diameter 7 mm, speed 10 mm/s and depression 4 mm, recorded in newton (N). Twenty fruit from different plants were taken at each stage, and each fruit was tested twice at equidistant points along the equatorial plane of the fruit. Values represent means ± standard error (SE; *n* = 20). For postharvest fruit firmness analysis, 20 fruit were used at each storage stage for measurement.

### Cytological assessment of fruit pericarp

Fruit epidermis was carefully isolated from the fruit surface with colourless nail enamel and tweezers. The detached segment was rinsed twice with distilled water before fixation on tissue‐bound slide and then mounted in 15% hydrochloric acid (HCl) under a cover slip. Observation was performed with the inner surface of epidermis on a Nikon microscope (Nikon Co. Ltd., Japan). Six exocarp slices from identical positions of different fruit were isolated at Br and Br+7 (RR) stages. For each exocarp slice, epidermal cell size (not including cell wall), cell separation and cell number (in one field of vision below a 40× object lens) were measured at three different positions using ImageProPlus software.

For cytological assessment of cuticle and fruit cells from exocarp to endocarp, three fruit of different plants were collected at the Br and Br+7 (RR) stages. Fresh pericarp sections were prepared and fixed in FAA solution (5% formaldehyde, 5% acetic acid and 50% ethanol) to examine pericarp cell wall structure. Fixed tomato fruit pericarp was dehydrated in ethanol series up to 95%, cleared with histo‐clear xylene and embedded in paraffin wax at 58 °C. Paraffin‐embedded transverse sections of 8 μm thickness were cut by microtome and attached to glass slides, and sections were dewaxed with xylene and stained with 0.05% toluidine blue. Micrographs of unstained fruit epicarp sections from RR tomato fruits were chosen for cuticle observation. A Nikon microscope was used, and cuticle thickness was estimated using ImageProPlus software

### Shelf‐life analysis

For determination of shelf‐life, fruit at the RR (Br+7) stage were detached and surface sterilized first, and then, each fruit was placed in a plastic jar kept at room temperature (23–25 °C and 55%–60% relative humidity). Every 5 day, fresh weight of each fruit was measured to calculate the PLW, and visual softening and collapse of fruit were assessed (Nambeesan *et al*., 2010). Twenty fruit were taken from different plants for analysis.

### *Botrytis cinerea* infection

The *B. cinerea* (B05.10) was grown on 1% potato dextrose agar, and conidia were harvested from sporulating colonies and filtered through glass wool as described by Stefanato *et al*. (2009). Surface sterilized fruit (Br+14) collected from WT and *SlPL*‐RNAi lines were sprayed thoroughly with spores (2.5 × 10^5^ spores/mL) five times in a fume cabinet and kept at 25 °C, in high humidity conditions. Infection symptoms were observed at 5 dpi. For wound inoculation, the fungal culture was diluted with sterile water to 5 × 10^4^ spores/mL and inoculated at 25 °C for 1.5 h to stimulate germination. Next, 5 μL of spore suspension was added to each wound. Lesion diameter was measured at 24, 48 and 72 h postinoculation. To quantify *B. cinerea* growth, 1 cm^3^ of fruit tissue around the lesion area was collected at 72 h postinoculation and total DNA extracted using a Plant Genomic DNA Kit (CWBIO, China). Purified DNA (50 ng) was used for qPCR as described by Zhang *et al*. (2013).

### Cell wall content analysis

For measurement of cellulose content, 100 mg of pericarp was ground with liquid nitrogen and digested with 60–70 mL cold 60% (V/V) sulphuric acid (H_2_SO_4_) for 1.5 h on ice. Next, filtration was performed and 2 mL of the filtered solution was mixed with 0.5 mL of 2% anthrone in a test tube, and concentrated H_2_SO_4_ was gently poured in along the tube wall. Subsequently, the reaction mixture was water‐bathed at 100 °C for 10 min and taken out for measurement at a wavelength of 620 nm, with pure cellulose used as a standard.

For analysis of hemicellulose content, hydrolysis of hemicellulose was conducted with a water‐bath at 100 °C for 45 min with 2 mol/L HCl. After centrifuging, the supernatant was transferred to a 100‐mL volumetric flask, which was then neutralized to rose pink using sodium hydroxide solution with phenolphthalein as the pH indicator. The solution was subsequently diluted and filtrated. Finally, the main content of hemicellulose, pentosan was measured using lichen phenol–HCl.

Cell wall polysaccharides were first prepared as alcohol‐insoluble solids (AIS) (Rosli *et al*., 2004). Afterwards, 50 mg of AIS was suspended in 50 mL of distilled water and incubated at 50 °C for 30 min under continuous stirring, and then, the solution was filtered and the residue washed thrice with 5 mL of distilled water. The pooled filtrate was denoted as WSP. The residue was extracted with 0.5 m H_2_SO_4_ at 100 °C for 1 h and denoted as HSP. Both WSP and HSP were analysed according to the m‐hydroxydiphenyl method (Blumenkrantz and Ashobe‐Hansen, 1973) using D‐galacturonic acid (D‐GA) as a standard. For each sample, tissue was pooled from six individual fruit. Three independent experiments were carried out and results expressed as mean ± SD of all replicates.

### Lipid peroxidation and antioxidant enzyme assays

Fresh fruit (Br+14) of *SlPL*‐RNAi and WT were homogenized and extracted with 0.1 mol/L (pH7.8) cold phosphate buffer saline on ice. Afterwards, the SOD activity was assessed according to Beauchamp and Fridovich (1971). POD activity was assessed by measuring the increase in absorbance at 470 nm due to the formation of tetraguaiacol (Sakharov, 1993). The CAT activity was measured according to Aebi (1974). MDA content was estimated according to Heath and Packer (1968).

### RNA‐Seq analysis for DEGs

RNA‐Seq was carried out by BGI Life Tech Co. Ltd. (Wuhan, China). Total RNA was isolated from Br+4 stage fruit using RNeasy Plant Mini Kit (Tiangen, China) following the manufacturer's protocol. Samples from both RNAi and WT fruit were performed with two biological replications. The cDNA libraries were constructed using a TruSeq™ RNA Sample Preparation Kit (Illumina) and subsequently sequenced using an Illumina HiSeq™ 2000 platform. The quality of RNA‐Seq data was assessed with the RSeQC‐2.3.2 program (http://code.google.com/p/rseqc/). Clean reads obtained from the raw reads were obtained by removing the adapter and low quality sequences and then mapped to the annotated genome sequence of *S. lycopersicum* in the Tomato Sol Genomic Network database (http://solgenomics.net/). Transcript abundance was also normalized by the fragments per kilobase of exon per million mapped reads (FRKM) method, and Cuffdiff software (http://cufflinks.cbcb.umd.edu/) was used to identify DEGs. A twofold difference in expression levels was set as the threshold for the determination of the significant changes. GO functional enrichment and KEGG pathway analysis were carried out with goatools (https://github.com/tanghaibao/goatools) and KOBAS (http://kobas.cbi.pku.edu.cn/home. do). All RNA‐Seq data were available in Data S2 online.

### Statistical analysis

All experiments were repeated three times independently, and all results are reproducible. Statistical results are presented as means ± standard error. To compare group differences, two‐tailed Student's *t*‐tests were used. *P* values less than 0.05 were recognized as significant.

## Supporting information


**Figure S1** Relative expression of *SlPL* and other highly homologous genes in WT and *SlPL*‐RNAi fruit.

**Figure S2** Relative expression of *SlPL* and *SlPL5* in WT and *SlPL*‐RNAi fruit with the inoculation of *B. cinerea* (B05.10).

**Table S1** Details of gene primers used in this article.
**Table S2** The DEGs involved in phytophormone metabolism and signaling transduction.
**Table S3** The DEGs encoding the members of TF families.
**Table S4** Expression analysis of DEGs involved in cell wall modification.
**Table S5** Expression analysis of DEGs involved in oxidative stress and pathogen resistance.
**Table S6** Evaluation of WT and RNAi plants for agronomic characteristics.

**Data S1** The accession numbers and according amino acid sequences of 22 tomato PLs, 26 *Arabidopsis* PLs and two softening‐related PLs from strawberry and banana.

**Data S2** The RNA‐Seq original data.
