# Supplementary figures and images for: Silencing of SlPL , which encodes a pectate lyase in tomato, confers enhanced fruit firmness, prolonged shelf‐life and reduced susceptibility to grey mould
Source: Plant Biotechnol J. 2017 May 16;15(12):1544–55. doi: 10.1111/pbi.12737 (PMC5698048; doi:10.1111/pbi.12737)

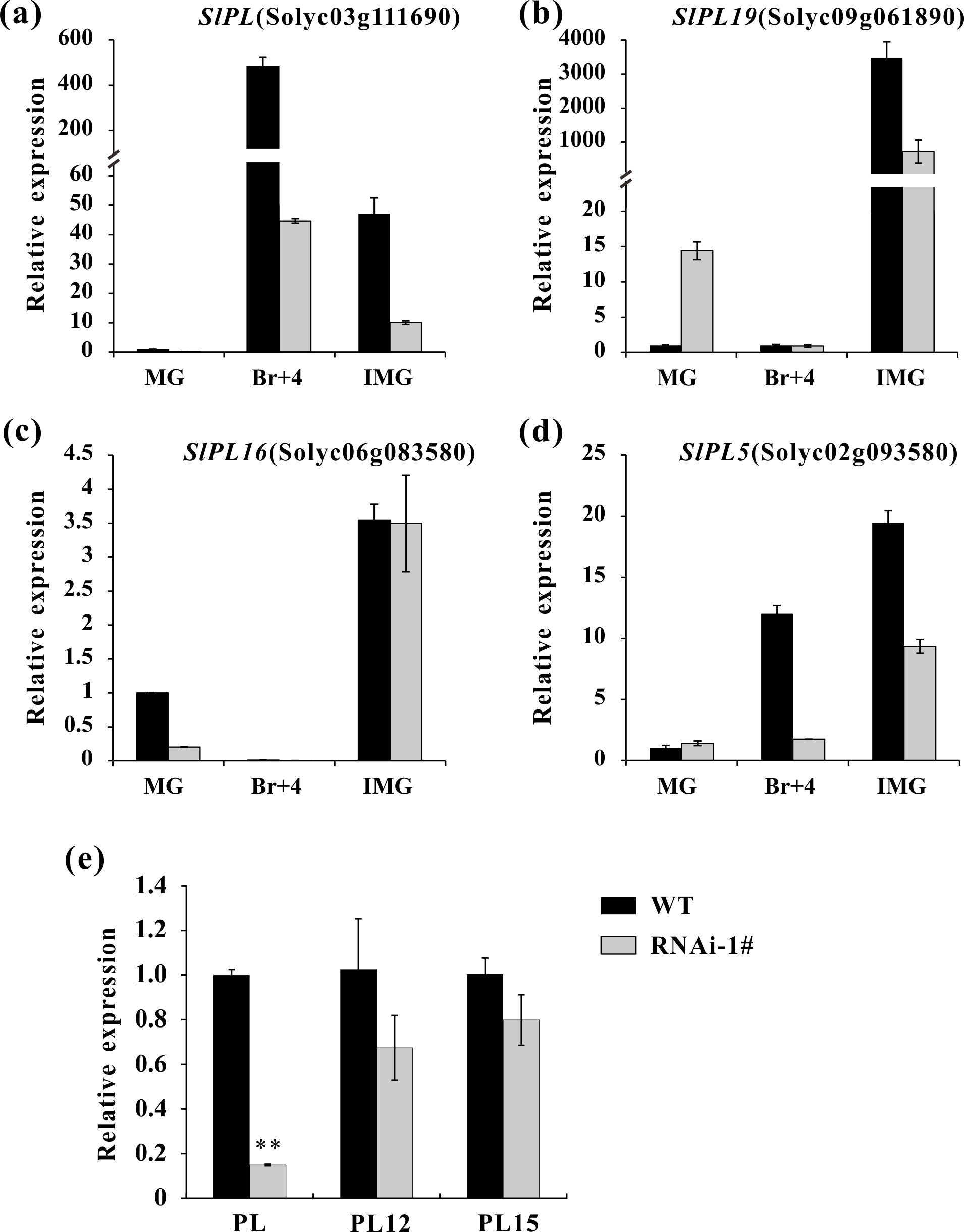

Supplement: Supplementary file 1 — Figure S1 Relative expression of SlPL and other highly homologous genes in WT and SlPL‐RNAi fruit. [file PBI-15-1544-s005.jpg]

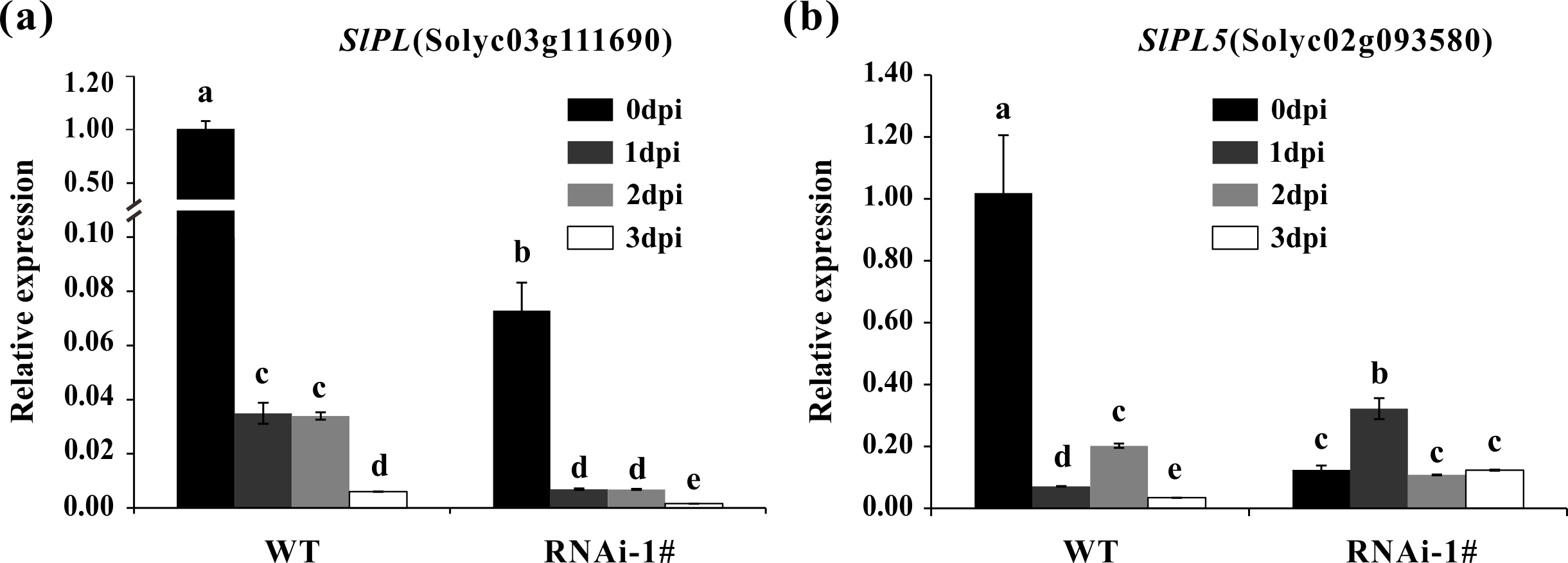

Supplement: Supplementary file 2 — Figure S2 Relative expression of SlPL and SlPL5 in WT and SlPL‐RNAi fruit with the inoculation of B. cinerea (B05.10). [file PBI-15-1544-s004.jpg]
