# Supplementary material for: Silencing of SlPL , which encodes a pectate lyase in tomato, confers enhanced fruit firmness, prolonged shelf‐life and reduced susceptibility to grey mould
Source: Plant Biotechnol J. 2017 May 16;15(12):1544–55. doi: 10.1111/pbi.12737 (PMC5698048; doi:10.1111/pbi.12737)
Supplement: Supplementary file 3 — Table S1 Details of gene primers used in this article. Table S2 The DEGs involved in phytophormone metabolism and signaling transduction. Table S3 The DEGs encoding the members of TF families. Table S4 Expression analysis of DEGs involved in cell wall modification. Table S5 Expression analysis of DEGs involved in oxidative stress and pathogen resistance. Table S6 Evaluation of WT and RNAi plants for agronomic characteristics. [file PBI-15-1544-s003.doc]

**Table S1. Details of gene primers used in this article**

| Name | Sequence (5’-3’) | Description |  |
| --- | --- | --- | --- |
| sPL-F | GGAATTCGGGTCTACAGCAATAACCAT  GGGGTACCGCACCATTCAACATTAGGTC  GCTCTAGAGGGTCTACAGCAATAACCAT  CGGGATCCGCACCATTCAACATTAGGTC  ACTGGGCACAACAGACAATCG  GCATCAGCCATGATGGATACTTT  TGTCCCTATCTACGAGGGTTATGC  AGTTAAATCACGACCAGCAAGAT  GCTGGAGCTTCTTCAAGTTATGC  GCAACCAAGTGCACCAGCATTC  AATCAGGAGCTGGAGCCTCTTC  GAACCTTTCCAACAACTCAGGG  CTCGGCCTGTTATTCTCAGC  GCTTGCCTCTCTCTAAAACCAA  AGTCACTTTGGATGGAGGACA  TGGCTAGACCCAAAAATGGA  CACGCACTGGGAGATGTATG  CCCTGGCTGTTAATTGTAGGA  AAGACGTTCATAGGAGCATCAAT  GATCCGCACGATAAATAGCC  ACAACTTTCCAACAAGGGAAGAT  TGTATGTTGCTATTCAGGCTGTG  ATTCCACAATATGGCATGAAATC  ATGTTATCTCATGTTATCTC  F: ACAATTCGATGCCAATTAATCTCC  R: GCAATTTGTTCGTCAGTGAGTCC  F: TGAACAACAAGACTCAGCTCCTG  R: CAAATAAGGTGCTCCATCCATGC  F: GGAGTAAGACAGAGACCATGGG  R: GCGGCGTCATCATAAGCTTTAG  F: GAAATAAGAGATCCACATAAAGCC  R: TGTGGTAACGATGACAATCTGGC  F: CGTTTATTTGGGACTCTTTGATAC  R: AAATTTGTAACTGCATCCTTCCCG  F: CGGTGGGTTAACGAGGTACAGG  R: GGTGAACATGGAATCCGAATCAG  F: GGTACTCCTCTATATATAGTTCCC  R: AGCTTGTGGTAATACGAAAGGGC  F: TTGGGAGCCTTGATGGAGC  R: GCCCTAAGGAACCATGATGTGA  F: TGAGCTCAAAGAAACACTAAGGG  R; TCTGTTCCATATTACTCCTAAGG  F: CTTGTTGTGAAAAAGTGGGCATC  R: ATCCGGCATTTTTAGGTAACGAC  F: GTCAGGTTATTGGAAGGCTACTG  R: ATGATCCAATCAGTCTTGACACC  F: GAAGAAGAACTAAAGCCGCCATC  R: GGAGCAATTAGGGGTAGATGGC  F: AATGTTATTCTCTGCTGCAATGG  R: ATATAAGATCTTCACTGAGCAACT  F: TATCAGATAAGATGTACGGACCC  R: TGTGAACATCTTGAGCTTTTCCC  F: GTATCGGCAGTGAGATGTCTGG  R: TTGACGATGTATCCACCTCTTCC  F: CATCATGTTACTAATCTCACCGG  R: TCAAATTCTTCAGCTGACTAAGG  F: GCAACAAATGGAATACCTTACCC  R: TGTGGGATTCTTGGAAGTTTGGG  F: GGCTCTATGTTACTTCTTGGACC  R: ATCCAGTTCCAACATAATTAGGC  F: TCAATTCGGGTGGTGTGGATCG  R: AAATCCCACCCAACATTCTGCG  F: TCTCTCTCCCAATTCAATTTGCC  R: GTTAAGTTACCTGTCTGTGTACC  F: TGCATCCTTTCCTAAACCAACCC  R: TCATTGTCTTGCCCTTATAATGG  F: GGTCAAAATCCAATCAAACTAGGG  R: CCACCATTAACTTGAACTCCTCC  F: AGCTACAACTCAACTAAAATCGC  R: CATATTTGCAACTTGAAAGAGGG  F: AGAGGCCAAGCTATAACTACGC  R: AATGAACCACCATCCGTTGTTGC  F: GAAGATGTGGGTTGATGAGAAGC  R: TTACAAGTTATAAAGTACCACCCG  F: AGCTCTTCATTCGCCTTGTTCC  R: TAGAAACCAACACCAAATTGACC  F: TGGTTCATTCACTGTCACAAGGG  R: GGGTAGCTAATGAATTCAGAATGC  F: AGCTCATTCTCCCTCCTCTTGG  R: CCCAACACCTGTATCTTGAATCC  F: TCTGAGGAGGAGTTGAAAGAGG  R: ATCACCATCAACATCAGCTTCCC | RNAi experiments  RNAi experiments  Positive plants selection  Housekeeping gene for qPCR analysis  qPCR analysis  qPCR analysis  qPCR analysis  qPCR analysis  qPCR analysis  qPCR analysis  Pathogen resistance experiments  Pathogen resistance experiments  qPCR verification for RNA-seq (ABA)  qPCR verification for RNA-seq (Auxin)  qPCR verification for RNA-seq (Ethylene)  qPCR verification for RNA-seq (Ethylene)  qPCR verification for RNA-seq (Ethylene)  qPCR verification for RNA-seq (bHLH)  qPCR verification for RNA-seq (bZIP)  qPCR verification for RNA-seq (GRAS)  qPCR verification for RNA-seq (MDAS-box)  qPCR verification for RNA-seq (MYB12)  qPCR verification for RNA-seq (NAC)  qPCR verification for RNA-seq (WRKY)  qPCR verification for RNA-seq (HSF)  qPCR verification for RNA-seq (EXP)  qPCR verification for RNA-seq (PG)  qPCR verification for RNA-seq (PGIP)  qPCR verification for RNA-seq (XTH)  qPCR verification for RNA-seq (CESA)  qPCR verification for RNA-seq (GH)  qPCR verification for RNA-seq (XIP)  qPCR verification for RNA-seq (SUS)  qPCR verification for RNA-seq (CYP)  qPCR verification for RNA-seq (HTC)  qPCR verification for RNA-seq (PR-1b)  qPCR verification for RNA-seq (PR-1a)  qPCR verification for RNA-seq (HSP90)  qPCR verification for RNA-seq (HSP90)  qPCR verification for RNA-seq (IRK)  qPCR verification for RNA-seq (CaM2) | |
| sPL-R |
| asPL-F |
| asPL-R |
| NPTII-F |
| NPTII-R  SlActin-F  SlActin-R  PL12-F  PL12-R  PL15-F  PL15-R |
| qSolyc03g111690-F  qSolyc03g111690-R  qSolyc09g061890-F  qSolyc09g061890-R  qSolyc06g083580-F qSolyc06g083580-R  qSolyc02g093580-F  qSolyc02g093580-R  SlACT-F |
| SlACT-R |
| BcCutA-F  BcCutA-R |
| Solyc08g005610.2.1 |
| Solyc09g065850.2.1 |
| Solyc04g071770.2.1  Solyc12g056590.1.1  Solyc02g093150.2.1  Solyc01g086870.2.1 |
| Solyc10g083380.1.1  Solyc07g052960.1.1  Solyc07g052700.2.1  Solyc01g079620.2.1  Solyc05g007770.2.1  Solyc01g079260.2.1  Solyc08g062960.2.1  Solyc01g112000.2.1  Solyc06g060170.2.1  Solyc07g065090.1.1  Solyc07g009380.2.1  Solyc08g082640.2.1  Solyc01g097270.2.1  Solyc01g098770.1.1  Solyc12g009300.1.1  Solyc03g115220.2.1  Solyc10g079570.1.1  Solyc00g174340.1.1  Solyc01g106620.2.1  Solyc03g007890.2.1  Solyc06g036290.2.1  Solyc01g104030.2.1  Solyc10g081170.1.1 |

**Table S2. The DEGs involved in phytophormones metabolism and signaling transduction.**

| Gene ID | log^2^ Ratio  (SlPel-1/WT-1) | | log^2^ Ratio(SlPel-2/WT-2) | | Description |  |
| --- | --- | --- | --- | --- | --- | --- |
| Auxin | |  |  | Auxin-repressed protein  Auxin-repressed protein  Auxin responsive SAUR protein  Auxin-responsive family protein  Auxin responsive protein  Nodulin-like protein  Auxin-regulated protein  Auxin-regulated protein  Nodulin-like protein  Auxin responsive protein  Aldo/ keto reductase family protein  GH3 family protein  Auxin efflux carrier family protein  Amino acid transporter  Late embryogenesis abundant protein  Ethylene responsive transcription factor 2b  24-sterol C-methyltransferase  ACC oxidase 3 (ACO3)  ACC oxidase 1 (ACO1)  Ethylene-responsive transcription factor 5  AP2-like ethylene-responsive TF  Ethylene-responsive transcription factor 4  AP2-like ethylene-responsive TF  Ethylene responsive transcription factor 2a  Ethylene-responsive transcription factor 5  Ethylene responsive transcription factor 1a  AP2-like ethylene-responsive TF  Ethylene-responsive transcription factor 10  Ethylene-responsive transcription factor 10  Ethylene responsive transcription factor 2b  AP2-like ethylene-responsive TF  Ethylene responsive transcription factor 2a  Multiprotein bridging factor 1  Ethylene-responsive TF CRF2-like  Ethylene responsive transcription factor 2b  Ethylene-responsive TF CRF2-like  Abscisic acid receptor PYL8  lycopene beta-cyclase  Glutamate receptor 3.4-like  LanC-like protein 2-like  Abscisic acid 8'-hydroxylase 1-like  Lysine decarboxylase-like protein  Gibberellin regulated protein  Gibberellin 2-beta-dioxygenase  F-box protein GID2  Gibberellin-regulated protein 2  GH3 family protein  Jasmonic acid 2  UDP-glycosyltransferase 79B6-like | | |
| Solyc01g099840.2.1 | | -2.49862 | -1.13001 |
| Solyc03g006360.2.1 | | -9.5812 | -7.88874 |
| Solyc03g082520.1.1 | | -2.3559 | -5.36057 |
| Solyc03g082530.1.1 | | -1.58595 | -6.02367 |
| Solyc03g120390.2.1 | | -1.18324 | -1.88238 |
| Solyc05g005860.1.1 | | -2.04821 | -2.67167 |
| Solyc06g030470.2.1 | | -1.46731 | -3.89375 |
| Solyc06g075690.2.1 | | -1.84924 | -5.00856 |
| Solyc08g075460.2.1 | | -2.89249 | -1.47545 |
| Solyc09g065850.2.1 | | -1.81319 | -4.01099 |
| Solyc09g097960.2.1 | | -2.68472 | -4.08937 |
| Solyc10g011660.2.1 | | -1.88994 | -1.32949 |
| Solyc12g095750.1.1 | | -2.0612 | -2.38042 |
| Solyc08g082080.2.1 | | 1.572938 | 1.562995 |
| Ethylene  Solyc01g095140.2.1  Solyc01g108240.2.1  Solyc01g111830.2.1  Solyc02g071380.2.1  Solyc02g071450.2.1  Solyc02g093130.1.1  Solyc02g093150.2.1  Solyc04g007000.1.1  Solyc04g049800.2.1  Solyc04g071770.2.1  Solyc04g078640.1.1  Solyc05g052050.1.1  Solyc07g018290.2.1  Solyc08g007820.1.1  Solyc08g007830.1.1  Solyc08g082210.2.1  Solyc11g072600.1.1  Solyc12g056590.1.1  Solyc01g104740.2.1  Solyc05g024230.1.1  Solyc09g075420.2.1  Solyc12g038800.1.1  ABA  Solyc01g095700.2.1  Solyc06g074240.1.1  Solyc07g052400.2.1  Solyc07g063320.2.1  Solyc08g005610.2.1  Cytokinin  Solyc04g081290.2.1  GA  Solyc11g011210.1.1  Solyc06g060800.2.1  Solyc07g047680.1.1  Solyc12g089300.1.1  JA  Solyc10g011660.2.1  Solyc12g013620.1.1  SA  Solyc04g079030.2.1 | | -4.13023  -7.44294  -1.89256  -2.66438  -1.26303  -1.30459  -2.15736  -3.05653  -2.66419  -1.16506  -1.22746  -1.26193  -8.45943  -6.74796  -8.633  -1.08377  -2.87992  -2.58802  1.899306  5.042712  2.166676  1.324944  -1.33243  -2.34013  -3.07039  -3.8305  -1.1615  -2.47917  -1.14933  1.015825  1.628817  1.520988  -1.88994  -2.31382  -2.550197 | -9.71605  -9.16742  -2.05346  -1.67101  -1.49023  -1.85656  -3.35252  -4.50779  -2.27085  -3.9429  -1.94641  -2.26957  -7.5157  -7.79269  -11.7035  -1.20507  -4.84337  -4.38047  2.243085  10.66178  1.164387  2.015597  -3.51759  -3.51715  -2.13245  -2.18253  -1.51333  -4.91144  -1.1401  1.051391  2.569895  1.880607  -1.32949  -2.47042  -2.29287 |

***Table S3. The DEGs encoding the members of TF families.***

| Gene ID | log^2^Ratio  (SlPel-1/WT-1) | log^2^Ratio  (SlPel-2/WT-2) | | Description |  |
| --- | --- | --- | --- | --- | --- |
| bHLH |  |  | Transcription factor bHLH130-like  Transcription factor bHLH13-like  Transcription factor bHLH144-like  Transcription factor bHLH13-like  Transcription factor bHLH3-like  Transcription factor MYC2-like  Transcription factor bHLH140-like  BZIP transcription factor  Bzip transcription factor  Z-box binding factor 2 protein  Unknown protein LOC101259007 isoform    Ethylene-responsive transcription factor 4  Transcription repressor TEM1-like  B3 domain-containing TF ABI3  B3 domain-containing Os11g0197600  AP2 transcription factor SlAP2c  Ethylene-responsive TF RAP2-7-like  Transcription repressor TEM1-like  AP2-like ethylene-responsive TF AIL5-like  AP2 transcription factor SlAP2d  Ethylene responsive transcription factor 2b  Ethylene-responsive transcription factor 5  Ethylene-responsive transcription factor 4  Ethylene-responsive TF RAP2-7-like  Ethylene responsive transcription factor 2a  Ethylene-responsive transcription factor 5  Transcription repressor TEM1-like  Ethylene responsive transcription factor 1a  AP2-like ethylene-responsive TFAIL5-like  Ethylene-responsive transcription factor 10  Ethylene-responsive transcription factor 10  Ethylene responsive transcription factor 2b  Ethylene responsive transcription factor 2a  Ethylene responsive transcription factor 2b  Scarecrow-like protein 32-like  MADS-box protein AGL62-like  MADS-box protein 1  MADS-box transcription factor 1  MADS box transcription factor  Agamous-like MADS-box protein  MADS-box transcription factor 1  MADS-box protein AGL66  MYB 12 transcription factor  MYB family transcription factor-like  MYB transcription factor  NAC transcription factor 29-like  NAC domain-containing protein 72-like  WRKY transcription factor 4  WRKY transcription factor 3  WRKY transcription factor 37  WRKY transcription factor 5  WRKY transcription factor 26  WRKY transcription factor 2  WRKY transcription factor 6  C2H2L domain class transcription factor  C2H2L domain class transcription factor | | |
| Solyc01g086870.2.1 | -1.71218 | -2.44453 |
| Solyc01g096050.2.1 | -1.42834 | -2.81969 |
| Solyc02g070880.1.1 | -1.2538 | -1.98847 |
| Solyc05g050560.1.1 | -1.34659 | -1.9277 |
| Solyc06g083980.1.1 | -1.12494 | -1.7156 |
| Solyc08g005050.2.1 | -1.01146 | -2.59432 |
| Solyc09g005100.2.1 | -3.27986 | -2.57175 |
| bZIP |  |  |
| Solyc02g073580.1.1 | -2.49476 | -6.48468 |
| Solyc09g009490.2.1 | -6.18239 | -6.80735 |
| Solyc09g009760.1.1 | -2.47805 | -2.40766 |
| Solyc10g083380.1.1 | -2.36283 | -1.44929 |
| B3 |  |  |
| Solyc04g007000.1.1  Solyc05g009790.1.1 | -3.05635  -1.53629 | -4.50779  -5.09755 |
| Solyc06g083600.1.1  Solyc07g054630.2.1  AP2  Solyc02g093150.2.1  Solyc04g049800.2.1  Solyc05g009790.1.1  Solyc07g018290.2.1  Solyc11g072600.1.1  ERF  Solyc01g108240.2.1  Solyc02g093130.1.1  Solyc04g007000.1.1  Solyc04g049800.2.1  Solyc04g071770.2.1  Solyc04g078640.1.1  Solyc05g009790.1.1  Solyc05g052050.1.1  Solyc07g018290.2.1  Solyc08g007820.1.1  Solyc08g007830.1.1  Solyc08g082210.2.1  Solyc12g056590.1.1  Solyc09g075420.2.1  GRAS  Solyc07g052960.1.1  MADS-box  Solyc01g106700.2.1  Solyc03g114840.2.1  Solyc04g078300.2.1  Solyc04g081000.2.1  Solyc06g064840.2.1  Solyc07g052700.2.1  Solyc07g052720.2.1  MYB  Solyc01g079620.2.1  Solyc12g006800.1.1  Solyc12g099120.1.1  NAC  Solyc05g007770.2.1  Solyc07g063410.2.1  WRKY  Solyc01g079260.2.1  Solyc01g079360.2.1  Solyc02g021680.2.1  Solyc02g072190.2.1  Solyc02g093050.2.1  Solyc03g116890.2.1  Solyc09g015770.2.1  C2H2  Solyc02g085580.2.1  Solyc10g084180.1.1 | -7.11894  -4.58095  -2.15736  -2.66419  -1.53629  -8.45943  -2.87992  -7.44294  -1.30459  -3.05653  -2.66419  -1.16506  -1.22746  -1.53629  -1.26193  -8.45943  -6.74796  -8.633  -1.08377  -2.58802  2.166676  -1.57805  -3.46259  -1.83328  -9.06878  -3.0376  -2.03019  -3.1633  -4.98793  -1.1235  -1.09427  -3.28864  -3.06298  -2.51608  -1.75069  -1.31657  -1.86307  -1.8951  -1.48505  -4.7874  -1.74015  -2.29546  -1.32193 | -6.14975  -2.42951  -3.35252  -2.27085  -5.09755  -7.5157  -4.84337  -9.16742  -1.85656  -4.50779  -2.27085  -3.9429  -1.94641  -5.09755  -2.26957  -7.5157  -7.79269  -11.7035  -1.20507  -4.38047  1.164387  -1.00421  -4.06402  -1.27096  -3.89616  -3.1092  -2.77923  -2.13425  -2.16993  -2.7211  -1.40361  -3.26199  -1.04271  -1.68777  -3.08544  -1.4718  -1.32685  -3.92168  -1.82045  -8.6865  -1.19784  -2.4018  -5.54314 |

***Table S4.*** ***Expression analysis of DEGs involved in cell wall modification.***

| Gene ID | log^2^Ratio (SlPel-1/WT-1) | | log^2^Ratio (SlPel-2/WT-2) | | Description |  |
| --- | --- | --- | --- | --- | --- | --- |
| cell wall modification  Solyc03g111690.2.1  Solyc02g093580.2.1  Solyc01g088590.2.1  Solyc01g102350.2.1  Solyc03g083620.1.1  Solyc03g083730.1.1  Solyc06g060170.2.1  Solyc03g123630.2.1  Solyc01g007940.2.1  Solyc01g098120.2.1  Solyc01g112000.2.1  Solyc02g090360.2.1  Solyc04g015620.2.1  Solyc05g009470.2.1  Solyc05g051780.2.1  Solyc06g063240.2.1  Solyc07g015860.2.1  Solyc07g062140.2.1  Solyc07g063880.2.1  Solyc08g067030.2.1  Solyc08g078670.2.1  Solyc08g079090.2.1  Solyc10g078910.1.1  Solyc11g071640.1.1  Solyc12g014270.1.1  Solyc03g123630.2.1  Solyc11g044910.1.1  Solyc01g098770.1.1 | -3.24395  -1.64909  -2.88287  -1.3689  -1.1969  -8.71081  -1.42884  1.962622  -10.7202  -1.25108  -1.66843  -1.08106  -1.52957  -1.26586  -2.07263  -1.04092  -2.35199  -1.43296  -1.67987  -2.24257  -1.66006  -1.11402  -1.64981  -1.27728  -3.79647  1.962622  1.066896  -11.8098 | -1.3251  -3.60204  -1.62075  -2.36595  -1.64793  -11.9155  -1.47256  1.62913  -9.35315  -1.42906  -5.76568  -6.04816  -2.14116  -1.99685  -4.07121  -1.47301  -4.66025  -1.63662  -1.4957  -3  -1.99139  -4.67945  -3.5869  -2.54286  -3.67025  1.62913  1.094729  -5.81057 | | Probable pectate lyase 18-like  Probable pectate lyase P18 precursor  Putative invertase inhibitor-like  Pectinacetylesteraselike protein  Pectinesterase  Pectinesterase  Probablepolygalacturonase-like  Pectinesterase  GGAT 2-like  Cell wall hydroxyproline  Expansin-like protein  Laccase-22  Os01g0611000 protein  Alpha-xylosidase 1-like  Gamma-glutamyltranspeptidase 1-like  Os03g0169000 protein  Peptide deformylase  Trehalose-phosphate synthase 1  Putative beta-glucosidase 41-like  Os01g0611000 protein  Aspartic proteinase nepenthesin-1-like  Monocopper oxidase-like protein  Os06g0207500 protein  Lysosomal beta glucosidase-like  Peptide-N4-asparagine amidase A-like  Pectinesterase  Beta-xylosidase 1  Xylanase inhibitor  Xylanase inhibitor  Endo-1 4-beta-xylanase  Probable polygalacturonase-like  Polygalacturonase inhibitor-like  Alpha-galactosidase  Xyloglucan endotransglucosylase 9  Xyloglucan endotransglucosylase 2  Xyloglucan endotransglucosylase 12  Xyloglucan endotransglucosylase 7  Cellulose synthase-like protein G3-like  Cellulose synthase A catalytic subunit 6  Cellulose synthase  Mannan endo-1,4-beta-mannosidase 7-like  Glucose-6-phosphate 1-epimerase-like  Alpha-xylosidase 1-like  Probable polygalacturonase-like  Lysosomal beta glucosidase-like  LeXET2 precursor  Putative beta-glucosidase 41-like  Endo-1 4-beta-xylanase  Lysosomal beta glucosidase-like  Endo-1,3-beta-glucosidase A precursor  Endo-1,3-beta-glucosidase B precursor  Wound-induced protein WIN2  Endochitinase  Endochitinase  Acid beta-fructofuranosidase  Endochitinase 3-like  Endochitinase  Endochitinase-like  Endo-1,3-beta-D-glucosidase precursor  Beta-D-xylosidase 1  Chitinase-like protein 1-like  O-acyltransferase WSD1-like  O-acyltransferase WSD1-like | | |
| Solyc09g065000.2.1  Solyc11g040330.1.1  Solyc06g060170.2.1  Solyc07g065090.1.1 | -2.23847  -3.56635  -1.42884  1.532242 | -1.86724  -3.08299  -1.47256  1.232155 | |
| Solyc03g019790.2.1  Solyc03g093080.2.1  Solyc07g009380.2.1  Solyc09g008320.2.1  Solyc12g017240.1.1  Solyc08g082640.2.1 | -1.95316  -1.6988  -2.21233  -1.49057  1.034865  -1.98248 | -2.27302  -4.94804  -1.61656  -1.41915  1.262464  -1.40846 | |
| Solyc11g005560.1.1 | -1.77861 | -2.79056 | |
| Solyc12g015770.1.1 | -2.5536 | -2.16993 | |
| Solyc02g084990.2.1 | -1.50771 | -1.36033 | |
| Solyc02g085100.2.1 | -2.06506 | -2.64922 | |
| Solyc05g009470.2.1 | -1.26586 | -1.99685 | |
| Solyc06g060170.2.1 | -1.42884 | -1.47256 | |
| Solyc06g076780.2.1 | -1.92719 | -1.12782 | |
| Solyc07g009380.2.1 | -2.21233 | -1.61656 | |
| Solyc07g063880.2.1 | -1.67987 | -1.4957 | |
| Solyc11g040330.1.1 | -3.56635 | -3.08299 | |
| Solyc11g071640.1.1 | -1.27728 | -2.54286 | |
| Solyc01g008620.2.1 | 4.545113 | 7.60733 | |
| Solyc01g060020.2.1 | 1.427771 | 1.597665 | |
| Solyc01g097270.2.1 | 1.709471 | 1.360036 | |
| Solyc02g082920.2.1  Solyc02g082930.2.1  Solyc03g083910.2.1  Solyc10g055800.1.1  Solyc10g055810.1.1  Solyc10g055820.1.1  Solyc10g079860.1.1  Solyc11g044910.1.1  Solyc12g098810.1.1  Solyc10g009430.2.1  Solyc01g095960.2.1 | 2.232383  1.071132  1.006683  1.942776  1.676792  2.675651  2.939148  1.066896  1.289108  -1.72511  9.778077 | 2.946643  1.164311  2.250147  1.070246  2.168529  2.641769  4.959661  1.094729  2.281236  -1.80583  4.74685 | |

**Table S5. Expression analysis of DEGs involved in oxidative stress and pathogen resistance*.***

| **Oxidative stress** | **Pathogen resistance** |
| --- | --- |
| Solyc02g071380.2.1; Solyc02g071450.2.1; Solyc03g080190.2.1; Solyc03g115220.2.1; Solyc03g122360.2.1; Solyc04g071800.2.1; Solyc05g047530.2.1; Solyc05g052240.2.1; Solyc05g052680.1.1; Solyc06g068270.2.1; Solyc07g043420.2.1; Solyc08g007210.2.1; Solyc09g011240.2.1; Solyc09g014280.1.1; Solyc09g015070.2.1; Solyc09g089740.2.1; Solyc09g089820.1.1; Solyc09g091510.2.1; Solyc10g052510.1.1; Solyc10g079570.1.1; Solyc12g005350.1.1; Solyc12g087980.1.1; Solyc02g077530.1.1; Solyc02g092250.2.1; Solyc03g111280.1.1; Solyc03g111290.1.1; Solyc03g111300.1.1; Solyc03g111710.2.1; Solyc03g112040.1.1; Solyc04g051730.1.1; Solyc04g078340.2.1; Solyc05g047530.2.1; Solyc05g052680.1.1; Solyc06g060190.2.1; Solyc07g006890.1.1; Solyc08g007210.2.1; Solyc09g014280.1.1; Solyc10g079570.1.1; Solyc12g087980.1.1 | Solyc01g060180.2.1; Solyc01g068080.2.1; Solyc01g105070.2.1; Solyc02g077530.1.1; Solyc03g096040.2.1; Solyc03g116910.2.1; Solyc04g064690.2.1; Solyc04g071800.2.1; Solyc04g071890.2.1; Solyc04g071900.2.1; Solyc05g047530.2.1; Solyc05g052680.1.1; Solyc06g076780.2.1; Solyc07g063880.2.1; Solyc08g007210.2.1; Solyc09g007920.2.1; Solyc09g014280.1.1; Solyc10g079570.1.1; Solyc11g044910.1.1; Solyc11g071640.1.1; Solyc12g007030.1.1; Solyc12g087980.1.1; Solyc00g174340.1.1; Solyc01g079260.2.1; Solyc01g079360.2.1; Solyc01g079620.2.1; Solyc01g087200.2.1; Solyc01g102880.1.1; Solyc01g104030.2.1; Solyc01g106620.2.1; Solyc01g106630.2.1; Solyc02g021680.2.1; Solyc02g070890.2.1; Solyc02g072190.2.1; Solyc02g072470.2.1; Solyc02g078530.2.1; Solyc02g079040.2.1; Solyc02g079540.1.1; Solyc02g088560.2.1; Solyc02g090430.2.1; Solyc02g090970.1.1; Solyc02g093050.2.1; Solyc02g094000.1.1; Solyc03g007890.2.1; Solyc03g116890.2.1; Solyc03g118810.1.1; Solyc03g119250.2.1; Solyc04g007490.2.1; Solyc04g009640.2.1; Solyc04g076460.2.1; Solyc04g077340.2.1; Solyc05g008310.2.1; Solyc05g009740.1.1; Solyc05g013220.2.1; Solyc05g051220.2.1; Solyc06g036290.2.1; Solyc06g068990.2.1; Solyc07g051860.1.1; Solyc07g051870.1.1; Solyc07g063750.2.1; Solyc07g063820.2.1; Solyc07g066550.2.1; Solyc08g076490.2.1; Solyc08g079460.2.1; Solyc09g007010.1.1; Solyc09g015770.2.1; Solyc09g075730.2.1; Solyc10g005300.2.1; Solyc10g008230.1.1; Solyc10g081170.1.1; Solyc11g008260.1.1; Solyc11g069590.1.1; Solyc12g005290.1.1; Solyc12g006850.1.1; Solyc12g009870.1.1; Solyc12g044190.1.1; Solyc12g096920.1.1; Solyc12g099120.1.1 |

**Table S6. Evaluation of WT and** RNAi plants for agronomic characteristics

| **Parameter** | **WT** | **RNAi** |  |
| --- | --- | --- | --- |
| Fresh weight of the aerial part (g, 30 days after transplanting) | 6.9124±0.5221  40.9821±1.5541  3.1291±0.3232  0.9671±0.0219  28.7728±1.8947  0.2209±0.0437  75.0653±7.3768  2.1973±0.2324 | 6.3145±0.2487  41.2564±1.8932  3.0148±0.2503  1.0205±0.0226  29.2339±2.0227  0.1809±0.0690 *  51.1269±4.5825 *  2.1447±0.3123 | |
| Days from anthesis to breaker(days) |
| Fruit weight (g)  Fruit weight/size(g·cm^-3^) |
| Fruit yield/plant (g)  100 seed weight (g)  Seed germination (%)  Pericarp width (mm) |

Values are mean±SEM(*n*=6)
