# Supplementary material for: Silencing of SlPL , which encodes a pectate lyase in tomato, confers enhanced fruit firmness, prolonged shelf‐life and reduced susceptibility to grey mould
Source: Plant Biotechnol J. 2017 May 16;15(12):1544–55. doi: 10.1111/pbi.12737 (PMC5698048; doi:10.1111/pbi.12737)
Supplement: Supplementary file 4 — Data S1 The accession numbers and according amino acid sequences of 22 tomato PLs, 26 Arabidopsis PLs and two softening‐related PLs from strawberry and banana. [file PBI-15-1544-s002.doc]

The accession numbers and according amino acid sequences used in Figure 1

SlPL1 (Solyc01g010740); SlPL2 (Solyc02g067450); SlPL3 (Solyc02g080910); SlPL4 (Solyc02g087670); SlPL5 (Solyc02g093580); SlPL6 (Solyc03g058890); SlPL7 (Solyc03g058910); SlPL8 (Solyc03g071570); **SlPL (Solyc03g111690)**; SlPL9 (Solyc03g113150);

SlPL10 (Solyc04g010230); SlPL11 (Solyc05g007080); SlPL12 (Solyc05g014000); SlPL13 (Solyc05g055510); SlPL14 (Solyc06g071020); SlPL15 (Solyc06g071840); SlPL16 (Solyc06g083580); SlPL17 (Solyc09g005850); SlPL18 (Solyc09g008380); SlPL19 (Solyc09g061890); SlPL20 (Solyc09g091430); SlPL21 (Solyc11g008140);

AtPLL1 (AT3G09540); AtPLL2 (AT3G55140); AtPLL3 (AT5G09280); AtPLL4 (AT4G22080); AtPLL5 (AT4G22090); AtPLL6 (AT1G11920); AtPLL7 (AT1G30350); AtPLL8 (AT1G14420); AtPLL9 (AT2G02720); AtPLL10 (AT3G01270); AtPLL11 (AT5G15110); AtPLL12 (AT5G04310); AtPLL13 (AT3G54920); AtPLL14 (AT5G55720); AtPLL15 (AT5G63180); AtPLL16 (AT1G67750); AtPLL17 (AT3G53190); AtPLL18 (AT3G27400); AtPLL19 (AT4G24780); AtPLL20 (AT3G07010); AtPLL21 (AT5G48900); AtPLL22 (AT3G24670); AtPLL23 (AT4G13210); AtPLL24 (AT3G24230); AtPLL25 (AT4G13710); AtPLL26 (AT1G04680);

FaPEL ([AAK66161.1](https://www.ncbi.nlm.nih.gov/protein/14531296?report=genbank&log$=prottop&blast_rank=2&RID=6P6SDB6U014)); MaPEL ([AAF19196.1](https://www.ncbi.nlm.nih.gov/protein/6606534?report=genbank&log$=prottop&blast_rank=1&RID=6P6WVY6G014))

>[Solyc01g010740](https://www.solgenomics.net/tools/blast/show_match_seq.pl?blast_db_id=224;id=Solyc01g010740.2.1;hilite_coords=331-806) (SlPL1)

MAFISGNSILLVLFIWLASLVPHLHARIAEFDPYLEKQALEALNSSLEAYTNNPEEITNAFNKEVGNALLKYKSLRRHLKEKDKCMATNPIDRCWRCDKNWAENRMDLEECARGFGHKTTGGKNGKYYVVTDESDDNVQEPKPGTLRHAVIQEEPLWIIFEKSMVIKLRQELMITSDKTIDGRGVAVHIAYGAGLMIQFVHNVIIHNIRIQNIISTNGGMIRDSINHIGLRTVSDGDGISIFGSNHIWIDHCTLSECTDGLIDAIMASTAITISNCKFNYHNDVMLLGATDAFPQDAIMQVTVAFNRFGEGLIQRMPRCRWGFFHVVNNDYTHWQMYAIGGSAHPTIISQGNRFKASDNPNTKQVTKRDYATESEWKKWQWVSEGDSFLNGAYFVESGPQDKKKTALTKNHKIKFKPGSHAGRLTRFAGVLKCKPGIPC

>[Solyc02g067450](https://www.solgenomics.net/tools/blast/show_match_seq.pl?blast_db_id=224;id=Solyc02g067450.2.1;hilite_coords=102-411) (SlPL2)

MTKSLFIIFFVLGAIIPAFNAHIGDFDEVWRRRAEEAIKFTHETYESEPANITLAFNQ

KTRHTVKELSTVVSKNETIRRALGTKKYDGPCTVTNPIDKCWRCDPNWADNRKKLVECSMGFGYKTTGGRDGEFYVVTDPSDDYTTPKPGTLRHAVIQKEPLWIIFEKNMKIKLHQELIMQGDKTIDGRGATVHITGGASIMIQYTKNVIIHGLHIHDIVEGSGGMVRDAVDHIGLRTKS

DGDGAAVAADNSTRRHLAKKYKGPCMATNLIDKCWRCDPQWADNREKYADCAMGFGSKATGGKGGRVYVVSDNSDSDVENPAPGTLRHAVIQTEPLWIIFERHMHIKLQRELLMQGHKTIDGRGFNIHIEKGAGLKMQGVSNVIISNLHVHNIVITPGGMIRDSAEHVGIRSEDEGDGISLFSATDIWIDHVSMSRATDGLIDAVKGSTGITISNCHFTDHDKVMLFGANDNHVEDKKMQITLAYNHFGKRLDQRMPRVRFGFFHIVNNDYTHWMRYAIGGNNGATIISQGNRFIAQASPLIKEVTHREKVEESEWKNWTWLSIDDDMQNGAFFKTSGDQDALSKLQDLNLIPAEPSYKVGIITKFAGSLACTAGRPC

>[Solyc02g080910](https://www.solgenomics.net/tools/blast/show_match_seq.pl?blast_db_id=224;id=Solyc02g080910.2.1;hilite_coords=2057-2351) (SlPL3)

MASLALVLALLFLCISTLQAEYYAPSKNYYAPSTTKKTMNVIDSCWRAKSNWAKNRYALADCAVGYGKAAIGGKNGAIYVVTNPSDDPVNPKPGTLRYGVIQSKPLWIIFNKDMVITLKNELMINSYKTIDGRGAKVEIAYGPCITIQRVSHVIIHGISIHDCKPGKRGIVRDSPVHAGHRNGADGDGIDIFQSTHVWIDHCYLARCTDGLIDVIHASTGVTISNNYFTQHDKVMLFGHNDNNKEDKIMKVTVAFNYFGPGLIERMPRVRLGYAHVANNRYEKWLMYAIGGSANPTIFSE

GNYFLASKSTQVTKRESKNGWQNWKWRSSKDKFLNGAYFIPSGYGTTNPYYSKAQSFPVADGSMVPSLTADAGPLRCTSYKSSKSSDKDETKSLHDFFPSYDPQPHKNGLLNVIDSCWRWKGDWSSNRKALADCAIGFGSSTIGGKYGDIYIVNDSSDDPINPKPGTLRYGAIQSEPLWI

IFKRDMVLTLENELMVNSYKTIDGRGAKVEISNGPCITLDYVTNVIIHGISIHDCKPGKK

GMVRSSPEHVGERSGSDGDAISVFTSSNVWIDHCYLARATDGLLDVIHASTAVTISNNYF

TEHDKVMLLGHNDEYTADRNMKVTVVYNHFGRELVQRMPRVRHGYAHVANNYYDQWLMYAIGGSADPTIFSEGNYFIAPDKAYNKEVTKRETEEKGWKSWKWRSSNDMFMNGAYFLPSGYGSIAPKYTRGQSFIVAHGAFTPSLTSNAGPLQCVVNEPC

>[Solyc02g087670](https://www.solgenomics.net/tools/blast/show_match_seq.pl?blast_db_id=224;id=Solyc02g087670.2.1;hilite_coords=1184-1672) (SlPL4)

MGGTKLVYLSLLLFSTFLVINGHIGEFDEVWRRRAQEADEWAIKAYKPDPINVTLAFAKETGQALKEIKEAKLAVNGTRRELKGGGKKYDGPCSVTNPIDRCWRCQPDWADNRKRLADCAMGFAKGTTGGKAGEIYVVTDSSDDTSDPKPGTLRYGVIQKEPLWIIFAKSMTIRLHQELIVQSDKTIDGRGVNVHIANGAGFMLQYVKNVIIHGLRIHDIVVGSGGMIRDAMDHVGQRTQSDGDGISIFGSSNIWVDHVSMWSCYDGLVDAIEGSTAVTISNSHFTDHNEVMLFGASDSSSIDQRMQITVAYNHFGKRLIQRMPRCRWGFIHVVNNDYTHWNMYAIGGSQHPTIISQGNRFIAPPDMFKKEVTKRDYSPESVWKQWSWRSQGDLFMNGAFFVESGDPDWTQKHIQLFDGVVSASGDQVTWITRFSGALNCKPGEAC

>[Solyc02g093580](https://www.solgenomics.net/tools/blast/show_match_seq.pl?blast_db_id=224;id=Solyc02g093580.2.1;hilite_coords=1-1509) (SlPL5)

MSTLFFTFSLLLLAPLLVISSIQDPELVVQDVHRSINASLTRRNLGYLSCGSGNPIDDCWRCNPNWEKNRQRLADCAIGFGKNAIGGKNGRIYVVTDSGNDDPVNPKPGTLRHAVIQDEPLWIIFKRDMVIQLKQELVMNSYKTIDGRGASVHISGGPCITIHHTSNIIIHGINIHDCKQSGNGNIRDSPNHSGWWDVSDGDGISIFGGKNIWVDHCSLSNCHDGLIDAIHGSTAITISNNYFTHHDKVMLLGHSDSFTQDKGMQVTVAFNHFGEGLVQRMPRCRHGYFHVVNNDYTHWEMYAIGGSAAPTINSQGNRFLAPNEKYRKEVTKHEDAPESQWRSWNWRSEGDLMLNGAYFRQTGAGASSSSTYARASSLSARPSSLVGSITTNAGPVNCKKGSRC

>[Solyc03g058890](https://www.solgenomics.net/tools/blast/show_match_seq.pl?blast_db_id=224;id=Solyc03g058890.2.1;hilite_coords=13-1501) (SlPL6)

MGGPKIKYSFLFLCITFATIIPSLMAHIGHYDEVWRRRAEEAKEYARKIYEPHPENVTLAFNQKLRDTMKELKKVKGTHNNSTRRGLGTKKYTGPCMVTNPIDKCWRCDPNWADNRKKLADCAMGFGSKAIGGKDGEFYVVTDNSDDYNDPKPGTLRHAVIQKEPLWIIFKRGMNIRLHQEMIMQSDKTIDARGVNVHITKGAGITLQYIKNVIIHGLHIHDIVEGNGGMVRDAVDHIGIRTKSDGDGISIFGASNIWIDHVSMQRCYDGLIDAVEGSTGITISNGHFTDHNEVMLFGASDSSSIDQVMQITLAFNHFGKRLIQRMPRCRWGYIHVVNNDYTHWNMYAIGGSMHPTIITQGNRFIAPPDIFKKQVTKREYNPESVWMQWTWRSEGNLFMNGAYFTESGDPEWSSKHKDLYDGISAAPAEDVTWMTRFAGVLGCKPGKPC

>[Solyc03g058910](https://solgenomics.net/tools/blast/show_match_seq.pl?blast_db_id=222;id=Solyc03g058910.2;hilite_coords=7-398) (SlPL7)

MEYSYRTKINVLFIVLILFVFAALGTAINAPRRKLTKKYRGPCMAVNSIDKCWRCDPFWAEDRQKMADCALGFGINAMGGKYGPYYIVTDNSDDDVVDPKPGTLRFGVIQKGPLWITFARSMRIRLTRELIVSSNKTIDGRGKYVHIANGAGIKIQSASNVIISNLRIHNIVPTAGGLLRESDDHLGLRGADEGDAISIFNSHDIWIDHISMSRATDGLIDAVAGSTNITISNCHFTDHEKVMLFGANDHAEEDRGMKITLAYNHFGKRLDQRMPRCRFGFFHLVNNDYTHWERYAIGGSSGATIISQGNRFIAEDKLLVKEVTYREKSTSSVEEWMKWTWITDGDDFENGATFTPSGDQNLLSKIDHLNLIQPEPSSKVGLLTKFSGALSCKIRRPC

>[Solyc03g071570](https://www.solgenomics.net/tools/blast/show_match_seq.pl?blast_db_id=224;id=Solyc03g071570.2.1;hilite_coords=62-723) (SlPL8)

MSSQSLFYRAKMPPATPILLFIYLILSFFSPLIDSQLNLTLPNQHPYPESVVHQLHRRVNESRLLFQTIVRDSKCHSTGNPIDDCWQCDPNWAKDRQRLADCSIGFGQSAMGGKGGQIYVVRDSSDRDTVNPIPGTLRHAVVQEEPLWIVFAADMVIKLKHELIINNYKTIDGRGANVHITGNGCITLQYVSHVIIHNVHIYNCVPSGNTNIRSSPTHVGWRGKSDGDGISIFGSHNIWIDHCALSHCTDGLIDAIMGSTAITISNNYFSHHDDVMLLGHDDKYLPDSGMQVTIAFNHFGEGLVQRMPRIRRGYVHVVNNDFTHWQMYAIGGSANPTINSQGNRYTAPDDPNLKEVTKREDTDNGQWDEWNWRTDGDTMVNGAFFVPSGQGLSNQYTKAYSVDPKSALLINQLTANAGVLGGPRDNSISILPQVGGGTSEGSGRGHDQSNSGSVDLFGMIFSGTGASAAAPPTTTPILFSFFILLTLYIITITTQLSKPFLHLL

**>**[**Solyc03g111690**](https://www.solgenomics.net/tools/blast/show_match_seq.pl?blast_db_id=224;id=Solyc03g111690.2.1;hilite_coords=399-1142) **(PL)**

**MGTSSVFLLFLLSFLLLLPSLLASSNPQQVVDEVHRSINGSRRNLGYLSCGTGNPIDDCWRCDPNWEKNRQRLADCAIGFGKNAIGGRDGKIYVVTDSGDDNAVTPKPGTLRHAVIQTEPLWIIFARDMVIQLKEELIMNSFKTIDGRGASVHIAGGPCITIQYVTNIIIHGIHIHDCKQGGNAMVRSSPSHYGWRTVSDGDGVSIFGGSHVWVDHCSLSNCKDGLIDAIMGSTAITISNNYMTHHDKVMLLGHSDTYTQDKNMQVTIAFNHFGEGLVQRMPRCRHGYFHVVNNDYTHWEMYAIGGSADPTINSQGNRFLAPDIRFSKEVTKHEDAPESEWKNWNWRTDGDLMLNGAFFTRSGVRTGSSSYAKASSLSARPSSLVANLVSSSGALNCKKGSRC**

>[Solyc03g113150](https://www.solgenomics.net/tools/blast/show_match_seq.pl?blast_db_id=224;id=Solyc03g113150.2.1;hilite_coords=1053-1614) (SlPL9)

MGLPKRNLFLFFVLVVLVVSIEAHIKEFDEVWKKRAQQAKKAARHAYNPNPKIVADHLNYQVDKAVRGSKSRRRDLQRYSGKCMATNPIDQCWRCDPNWARNRMKLTDCVLGFGRKTTGGKGGKIYVVMDNSDNELVNPKPGTLRHAVIQPEPLWIIFAKNMVIKLNQELIMTSNKTIDARGRQVHIAHGGGLMLQFIHNVIISNLHIHDTKAGAGGLIRDSVSHYGYRSKSDGDGISIFGSTNVWIDHISMSNCQDGLIDAVEGSTAITISNCHFTKHNDVMLFGASDTASGDSVMQITLAFNHFGHGLTQRMPRVRWGFVHVVNNDYTHWLMYAIGGSMHPTILSQGNRFIAPPNPNAKEVTKRDYAPENVWKNWVWKSQGDLMMNGAFFVESGDPKHAFLKGPDMITSKPGSSVSSLTQFSGSLKCIEGRPC

>[Solyc04g010230](https://www.solgenomics.net/tools/blast/show_match_seq.pl?blast_db_id=224;id=Solyc04g010230.2.1;hilite_coords=62-1548) (SlPL10)

MAALPYADVDSSLKALAGRAEGFGRFAIGGLNGPVYSVTTLADDGPGSLRDGCRKKEPLWIVFEVSGTIPLTSYLRVSSHKTIDGRGQRIILTGKGLQLKDCEHIIVCNLEFEGGRGHDVDGIQIKPNSRHIWIDRCSLRDYDDGLIDITRQSTDITISRCYFAQHDKTMLIGADPSHVGDRCIRVTIHHCFFDGTRQRQPRVRFGKVHLYNNYTRNWGIYAICASVESQIYSQCNIYEASQKKKAFEYYTEKAADKEEARSGLIRSEGDMFLNGAQGSLLTGIGGECVFHPSEFYPVWTLEPASDSLKGILHICTGWQSVSLPQEECARQLKPAR

>[Solyc05g007080](https://www.solgenomics.net/tools/blast/show_match_seq.pl?blast_db_id=224;id=Solyc05g007080.2.1;hilite_coords=26-508) (SlPL11)

MASISFNNSVIFLFSLFCFSSIIPKLYANIADFDPYLEKRAEEALQSSLAAYNENPEGVTQIFNKEVGETLLNGTRRHLKEKDKEDKNDKDDKEGDEKSVDGCKAYNPIDKCWRCDKNWANNRKALADCARGFGHGTTGGKDGRFYVVTDPSDNNVEEPVPGTLRHAVIQEEPLWIIFEKSMIIRLKQELMINSNKTIDGRGVSVHVAYGGGLTLQFVHNVIVHNIRVHHILSKNGGMIRDSVKHIGLRTVSDGDAISLFGANRIWIDHCTLTKGADGLLDAIMASTAITISNCKFNHHNDVMLLGANDAFPQDKIMQVTVAFNRFGKGCIQRMPRCRWGFFHVVNNDYAKWEMYAIGGTANPTIISQGNRFKAADNPNTKEVTNRNGAPEALWRNWQWRSEGDLFKNGAFFRESGPEIKSTPFTEHTTIQFEPAKFVGRLTRKAGVIQCKIGKAC

>[Solyc05g014000](https://www.solgenomics.net/tools/blast/show_match_seq.pl?blast_db_id=224;id=Solyc05g014000.2.1;hilite_coords=21-1509) (SlPL12)

MGMPLSFLLLLTLLSPIFTFSSHVPDPEVIVQQVNEKINASRRNLGYLSCGTGNPIDDCWRCDPNWEKNRQRLADCAIGFGKQAIGGKDGKIYVVTDTSDDPVNPKPGTLRYGAIQDEPLWIIFSRDMVIKLKEELMLNSFKTIDGRGASVHIAGGPCITIQYVTNIIIHGLNIHDCKQGGNAYVRDSPQHYGWRTISDGDGVSIFGGSHVWVDHCSLSNCNDGLIDAIRGSTAITISNNYMTHHNKVMLLGHSDSFTRDKNMQVTIAFNHFGEGLVQRMPRCRHGYFHVVNNDYTHWEMYAIGGSASPTINSQGNRFLAPNDIFNKEVTKHEDAAESEWKNWNWRSEGDLMLNGAFFIRSGAGASSSYAKASSLSARPSTLVNSITMNAGALGCKKGKRC

>[Solyc05g055510](https://solgenomics.net/tools/blast/show_match_seq.pl?blast_db_id=222;id=Solyc05g055510.2;hilite_coords=38-418) (SlPL13)

MISLTFILSFFFFLILSFSSLITSTFNNQTLSHQHPFPESVVQQVNRRINESISRRQISDTTVINYQCLTGNPIDDCWRCDPNWVDNRQQLADCAIGFGHGAVGGKGGRYYLVSDPSDYDTVNPTPGTLRHAVIQDEPLWITFAGDMIIRLKHELMINNYKTIDGRGANVHVTGGGCITLQYVTNVIIHNIHVYNCIPSGNSNIRQSTTQVGWRGMSDGDGISIYSSRNIWIDHCALSHCTDGLIDAIMGSTAITISNSYFTHHDKVMLLGHDDRYVPDVGMQVTIAFNHFGEGLVQRMPRCRRGYIHVVNNDFTEWQMYAIGGSANPTINSQGNRFTAPEDPNAKEVTKRVDVDERDWTEWNWRTEGDEMVNGAYFVPSGDGISNQYALASSMEPKSAFLIEQLTMNAGVIGVPRDTTVAMSFGGRTRTTIAANRSSSVRPSRSKDGDGGFLEKVFGSVASAGSSTSSPSSSTITILFSLLILYIITNNIGLL

>[Solyc06g071020](https://www.solgenomics.net/tools/blast/show_match_seq.pl?blast_db_id=224;id=Solyc06g071020.2.1;hilite_coords=1-554) (SlPL14)

MVTLINRSSFFVILCTLFFSIEANIGEFDEVWRTRATQANKNAKESYNPNPEKVAANFNKHVHRSEEGSNSTRRDLHKYNGPCVATNPIDRCWRCDPHWAKNRQKLADCVLGFGHHTTGGKGGKIYEVTDPGDTDMVNPKQGTLRHAVIQPGPLWIIFAHHMVIKLNQELIMTGDKTIDARGQQVHITGGASLMLQYINNVIIHGLHIHDIKAGNGGLIRDSINHYGFRTKSDGDGISIYGSTNIWIDHVSMSNCDDGLIDAVQASTAITISNCHFTHHNDVMLFGASDSFKQDEILQITLAFNHFGQGLIQRMPRVRWGFVHAVNNDYTHWLMYAVGGSQQPTILSQGNRFIAPPNPNAKEVTKREYSPESVWKNWVWRSQGDLMMNGAFFVESGDPNHKFTTGPDMIHPRAGSDAGRLTRFSGSLNCIEGKPC

>[Solyc06g071840](https://solgenomics.net/tools/blast/show_match_seq.pl?blast_db_id=222;id=Solyc06g071840.1;hilite_coords=28-359) (SlPL15)

MATSSISLIFLLSFLLLIPSLLASSPLQNHQYVVDQVDRSINVSRRNLGYLSCGTGNPIDDCWRCDPNWEKNRQRLANCAIGFGKNAIGGRDGKIYVVTNSGNDDPVNPKPGTLRYGVIQDEPLWIIFASDMVIQLKEELIMNSFKTIDGRGANVHIAGGPCITIQYVSNVIIHGIHIHDCKQGGNAMVRSTPQHYGWRTISDGDGVSIFGGSDIWIDHCSLSNCVDGLIDAIMGSTSITXSNNYMTNHDKVMLLGHSDSHVQDKNMQVTIAFNHFGEGLVQRIPRCRHGYFHVVNNDYTHWEMYAIGGSANPTINSQGNRFLASDIRFSKEVTKHENAPESEWKNWNWRSDGDLMLNGAFFVKSGAGASSNYAKASSLSAKSSSLISSLVSGAGALSCWKGSRC

>[Solyc06g083580](https://solgenomics.net/tools/blast/show_match_seq.pl?blast_db_id=222;id=Solyc06g083580.2;hilite_coords=60-433) (SlPL16)

MAAALRLSCLCFLQLLIVIVLFINSSADNSIEQAEQFQSLSNSTMTNRLSGNDELRHEHAEDDPEAIASMVDMSIRNSTERRKLGYFSCRTGNPIDDCWRCDRNWQRNRRRLADCSIGFG

RNAIGGRDGRFYVVTDSGDDDPVNPRPGTLRHAVIQDEPLWIVFKRDMHITLKQELIMNS

FKTIDARGVNVHIANGACITIQFVTNIIIHGLHIHDCKPTGNALVRSSPSHYGWRTMADG

DAISIFGSSHIWVDHNSLSNCADGLVDAIMGSTAITISNNYFTHHNEVILLGHSDTYVRD

KVMQVTIAFNHFGEGLIQRMPRCRHGYFHVVNNDYTHWEMYAIGGSAAPTINSQGNRYLAPVNPFAKEVTKRVEPWEGSWRQWNWRSVGDLLLNGAYFTASGRAAPGSYARASSLAAKSSSLVGMITSNAGALS

>[Solyc09g005850](https://www.solgenomics.net/tools/blast/show_match_seq.pl?blast_db_id=224;id=Solyc09g005850.2.1;hilite_coords=2-1526) (SlPL17)

MGNHHGRFSHSKHHHHHHNQQGPPFFVPTQQTQNTQMGIALPYANVDSNLRGLAGQAEGFGGSSIGGRDGHVYQVLNLNDDGPGSLRDGCRKKEPLWIVFEVSGTIELRSHLSVSSFKTIDGRGQKIKLTGKGLRLKECEHVIICNLEFEGGRGHDVDGIQIKPKSRHIWIDRCSLSDYDDGLIDITKESTDITVSRCHFSKHDKTMLIGGDSSNCGDRCMRVTIHHCFFDGTRQRHPRVRFAKVHLYNNYTRNWGIYAVCASVESQIYSQCNIYEAGQKKVAFKYLTEKAADKEEACTGSIKSEDDLFVCGTQAGLLSTCSENNVFNPSEFYQTWTVERPSDDLKHYLQHCTGWQCIPRPN

>[Solyc09g008380](https://www.solgenomics.net/tools/blast/show_match_seq.pl?blast_db_id=224;id=Solyc09g008380.2.1;hilite_coords=1-647) (SlPL18)

MFQISYIVVFLILTSFFPLGFGIFLNVTTLPGQHPDPESVALEVNRKVNASLSIFQSRRKMLSYTQSSCQTGNPIDDCWRCDHSWQLNRQRLADCAIGFGQYALGGKGGRYYVVTSSSDPDPVDPPPGTLRYGVIQEEPLWIVFSASMEIKLSEELIFNSHKTLDGRGVNVHITGGGCITLQYISNVIIHNIHVHHCYESGDTNVRSSPTHFGYRGKSDGDGISIFGSRDIWIDHCSLSNCKDGLIDVVMGSTGITISNNHFSHHNEVMLLGHNDDYLPDSGMQVTISFNHFGKKLIQRMPRCRRGYIHVVNNDFTRWEMYAIGGSGNPTINSQGNRYIAPFDPFAKEVTKRVDTDEGKWRNWNWRSEGDVMANGAYFVASGEEVEIKYEKAYSVEPKSADFIDQITLNAGVLIHRGSNSGKWTATTNNDTESAGDDGGGEDLVAISGDSDDDYGGDEESRSSTIYSNFSLLFNLLMALLALL

>[Solyc09g061890](https://www.solgenomics.net/tools/blast/show_match_seq.pl?blast_db_id=224;id=Solyc09g061890.2.1;hilite_coords=271-1002) (SlPL19)

MAIYWRSCLSLSALLILVLLVINVNPSHSAEEAEQFQSLKNLTMTNSLSEENVPNHEHAV

DDPEKVVSMVDMSIKNSTERRKLGFFSCGTGNPIDDCWRCDRNWQRNRKRLADCAIGFGRNAIGGRDGKYYVVTDPNDDDPVNPRPGTLRHAVIQDRPLWIVFKRDMVITLKQELIMNSFKTIDGRGVNVHIANGACITVQFVTNIIIHGINIHDCKPTGNAMVRSSPSHFGWRTIADGD

GISIFGSSHIWVDHNSLANCADGLIDAIMGSTAITISNNYFTHHNEVMLLGHSDSYVRDK

IMQVTIAYNHFGEGLIQRMPRCRHGYFHVVNNDYTHWEMYAIGGSASPTINSQGNRYLAPANPFAKQVTHRVEQSDVWKHWNWRSEGDLMLNGAFFTPSGHGAAASYARASSLAAKASSLVGTLTSNAGALTCRRGYQC

>[Solyc09g091430](https://www.solgenomics.net/tools/blast/show_match_seq.pl?blast_db_id=224;id=Solyc09g091430.2.1;hilite_coords=5-1784) (SlPL20)

MAMASKKWLSFSFSFLLILLLLVGVYAAGVQQSNDGSDTRTVEKEQLLSSENSTMAVSLEDVEEKLSKHAVDDPEEVVAMVAQSIRNSTERRKLGYFSCGTGNPIDDCWRCDPNWQKNRKRLADCGIGFGRNAIGGRDGRYYVVTDNRDDDPVNPRPGTIRHAVIQEEPLWIVFSRDMVIQLKQELIMNSFKTIDARGYNVHIANGACLTIQFVTNIIIHGLHIHDCKPTGNAMVRSSTTHFGWRTMADGDAISIFGSSHVWVDHNSLSHCADGLVDAVMGSTAITISNNHFAHHNEVMLLGHSDSYTKDKQMQVTIAYNHFGEGLIQRMPRCRHGYFHVVNNDYTHWEMYAIGGSANPTINSQGNRYLAPANRFAKEVTKRVDTAAGQWKGWNWRSSGDLMLNGAYFTPSGAGASASYARASSLGAKSSSMVGAITSSAGPLACRRSRTC

>[Solyc11g008140](https://www.solgenomics.net/tools/blast/show_match_seq.pl?blast_db_id=224;id=Solyc11g008140.1.1;hilite_coords=1-1223) (SlPL21)

MFSLICILLFCLLVSFFPQIIASFNLTLPHQHPYPEVVVQEVQRKVNESISRRQLWNTTM

NAITTQCETGNPIDDCWRCDPNWSKNRQRLADCAIGFGQNAIGGKNGKYYVVSDSSDLDTVNPTQGTLRHAVIQEEPLWIIFSGDMFIKLKHELIVNSYKTIDGRGAKVHITGNGCITLQ

YISNVIIHNIHIYNCLPSGNTDIRSTPTHVGHRGRSDGDGISIFSSRNIWIDHCALSHCTDGLIDAIMGSTGITISNSYFSHHDEVMLLGHDDSYSPDAGMQVTIAFNHFGVGLVQRMPRCRRGYIHVVNNDFTEWQMYAIGGSANPTINSQGNRYTAPVDANAKEVTKRVETDEGEWSG

WNWRTDGDIMVNGAFFVPSGEGLSNQYAKASSVEPKSAAIIDQLTLNAGVFGATSESVDQIEDSVNSLKAKEYTLSISIFLVYFLFSSV

> AT3G09540 (PLL 1)

MGNLHGIHRSHHGGSNFPGEAPNSPFPPPYTAAPAPAPAPSSPSDHHMTVGPYCHVDSSLRSLAGKAEGFGRAAVGGLNGPICHVTSLADEGPGSLREACKRPEPLWIVFDVSGTINLSSFVNVSSHTTVDGRGQKVKITGKGLRLKECENVIICNLEFEGGVGPDADAIQIKPKSHNIWIDRCSLKNYYDGLIDITRESTDITVSRCHFMNHNKTMLIGADTSHVTDRCIRVTIHHCFFDGTRQRHPRVRFAKVHLFNNYTRHWAIYAVGAGVESQIHSQCNIYEAGEKKTVFKYITEKAADKEKPGAGFVRSEGDLLLNGAKSCLSQGGERYVFSPIQHYSEWTVESPTDILKNYLKHSTGWQNLPLPLDRPPTTA

> AT3G55140 (PLL 2)

MTSLPYADADCSLRALAGRAEGFGRFAVGGLHGDLYVVTSLADDGPGTLREGGRRKEPLWIVFAVSGTINLNSYLSVSSYKTIDGRGQRIKLTGKGIRLKECEHIIICNLEFEGGRGHDVDGIQIKPKSRHIWIDRCSLRDYDDGLIDITRQSTDITVSRCYFAQHDKTMLIGADPSHVEDRCIRVTIHHCFFDGTRQRQPRLRFGKVHLYNNYTRNWGIYAVCASVEAQVFSQCNIYEAGVKKKTFEYYSEKAADKEEARTGLVRSENDLFLNGAQPSLMTGASEECVFHPSEHYPTWTVEPPSETLKQIMQICTGWQSLSRPSDHGVPK

> AT5G09280 (PLL 3)

MTGNIGKGVTQYKVTDPSDDPLNPKPGTLRYGATLVKGKKWITFKRNMKIKLHKPLLISSFTALDGRGASVHISGPACLIVYRATDVIIHGLKIHDCKAHPPSSVMGPDSKIIQLGHMDGDAIRLVTAKKVWIDHNTLYDCEDGLLDVTLGSTDVTVSNNWFRNQDKVMLLGHDDGYVKDKDMRVTVVFNHFGPNCNQRMPRVRHGYAHVANNYYQGWTQYAIGGSMSPRVKSESNYFVAPKSGSKEVLEKHGPITAQIKGSQSQTLNRLKYSLLQPERYTAPETPSVSTTTLNMTF

> AT4G22080 (PLL 4)

MTLFTVSCLLVVLFLCHSLVHAENNGYYGYTPTVANYLPEKPQNIMNPVDSCWRLKSDWAANRKDLADCVVGFGSSTLGGKKGNLYVVTNPYDNAQNPQPGSLRYGVIQAKPLWITFAKDMVITLENELMVNSYKTIDGRGAKVEIAYGPCITIQDVTNVIVHGISIHDCKPGKYGMVRSSPTHVGHRKGSDGDAIAIFGSSNIWIDHCYLASCTDGLIDVIHASTGITISNNYFTQHDKVMLLGHNDDFVQDVKMKVTVAFNHFGPGLVERMPRVRRGYAHVANNRYDKWIMYAIGGSADPTIFSEGNYFIASDKSNSKEVTKREVKGGWNNWRWRTSKDVFKNGAYFVPSGYGSISLPYSSAQRFTVAPGNLVPSLTADAGPLNCNRNGPCY

> AT4G22090 (PLL 5)

MTHFTVSCLLVALFLCQSLVHAAYNGYYGYSPAAAPYPAEEPQNIMNPVDSCWRLKSDWDVNREDLADCAVGFGSSTLGGKKGNIYVVTNPYDNAQNPHPGSLRYGVIQAKPLWITFAKDMVITLANELMVNSYKTIDGRGAKVEIAYGPCITIQDVTNVIVHGISIHDCKPGKSGKVRSSPTHVGHRKGSDGDAITIFGSSNVWIDHCYLASCTDGLIDVIHASTAITISNNYFTQHDKVMLLGHNDNFVKDVKMKVTVAFNHFGPGLVERMPRVRRGYAHVANNRYDKWIMYAIGGSADPTIFSEGNYFIASDKSYSKEVTKREVKGGWNNWRWRTSNDVFKNGAFFVPSGYGSIPLPYSSAQRFTVAPGNLVPSLTADAGPLNCNRNGPCY

> AT1G11920 (PLL 6)

MASLFLTIISLLFAAFSSSVVEAAYSNGYTIPKLLPNPIDSCWRRNPYWASNRRALADCAVGFGKSAVGGKYGSIYVVTNPSDDPENPRPGTLRYAVIQSKPLWITFARDMVIVLRNELIMNSYKTIDGRGAKVEIAYGPCITIQHVSHVIIHGISIHDCKPGKSGRVRSSPTHVGSRKGSDGDAIAIFDSSHIWIDHCFFSRCQDGLIDVLHASTAVTISNNYFTQHDKVMLLGHNDNNVEDKIMRVTIAFNHFGPGLIERMPRVRRGYAHVANNRYEKWQMYAIGGSADPTIFSEGNYFVASDDPSKKQVTKRIDSGYDWKRWKWRTSKDVFKNGAYFVPSGYGTVTPLYGRAERFPVSHGSLVPLLTSSAGPLHCYSGRIC

> AT1G30350 (PLL 7)

MASLVVIVSLLLAAFASPLLETAHSYNVTAPRVSLNPIDACWRRNPKWATNRQALAHCAVGYGKAAIGGKNGPIYVVTNPSDNPTRPSPGTLRYAVSQPKPLWITFARDMVIVLKSQLMINSYKTIDGRGAKVEIANGPCLRIRQVKHVIIHGISIHDCKADPNGMDGDGIRVFQSTHVWIDHCFLSRCHDGLIDVIVSSTAVTISNNYFTQHDKVMLLGHDDSYMGDKDMRVTIAFNTFGPGLIERMPRVRRGYAHVANNRYEKWQMYAIGGSANPIIFSEGNYFVAPEKRSSKQVTKRMMAGPDSKRWKWGTSRDVFMNGAFFGPPGVIVRPLYKGGEGFRVAHGSLVPSLTSSAGPLRCYVGRIC

> AT1G14420 (PLL 8)

MAAAFLNLGGYVFVFFSSFLAIVAPQVRGNVAVFDSYWTQRQSDALKQTIGSYDPHPLNVTNHFNYHVNIAVDASESRNDTRRELTQVRSGRKTHKSSGKCLAYNPIDNCWRCDRNWANNRKKLADCVLGFGRRTTGGKDGPIYVVKDASDNDLINPKPGTLRHAVTRDGPLWIIFARSMIIKLQQELMITSDKTIDGRGARVYIMEGAGLTLQFVNNVIIHNIYVKHIVPGNGGLIRDSEAHIGLRTKSDGDGISLFGATNIWIDHVSMTRCADGMIDAIDGSTAVTISNSHFTDHQEVMLFGARDEHVIDKKMQITVAFNHFGKRLEQRMPRCRYGTIHVVNNDYTHWEMYAIGGNMNPTIISQGNRFIAPPNEEAKQITKREYTPYGEWKSWNWQSEGDYFLNGAYFVQSGKANAWSSKPKTPLPNKFTIRPKPGTMVRKLTMDAGVLGCKLGEAC

> AT2G02720 (PLL 9)

MVNLGSYVFVFVALSLTVVVPSVQAHIAEYDEYWTQRQTNALRETLESYDPNPENVTDHFNYHAALAMETTGIVNETRRDLRQVGRGKKTTRRGGRFESLNAIDKCWRGDKNWDKNRKKLADCVLGFGRKTTGGKNGPIYVVTDPSDNDLLKPKPGTIRHAVTRDRPLWIIFARSMIIKLQQELIITNDKTIDGRGAKIYITGGAGLTLQFVRNVIIHNIHIKQIKRGAGGLIIDSEQHFGLRTVSDGDGINIFGATNVWIDHVSMTDCSDGMIDAIMGSTAITISNSHFTDHDEVMLFGGTNKDVIDKKMQITVAFNHFGKRLKQRMPRVRFGLVHVVNNDYTHWEMYAIGGNMNPTIISQGNRFIAPPIEDSKQVTKREYTPYPEWKSWNWQSEKDYFLNGAYFVQSGKANAWSATPKNPIPRKFAIRPQPGTKVRRLTKDAGTLGCKPGKSC

> AT3G01270 (PLL 10)

METARLFKLVCVICIASLIPTIRANVADETDEYWVNKANEARKHTLMAYHPDPYEIVDHFHERHYDNSTDVEGTEEEKAVASEEEDVIEMISSPTNSTRRSLTGRGKGKGKGKWSKLTGPCTASNPIDKCWRCQPDWARRRKKLVHCVRGFGYRTTGGKRGRIYVVTSPRDDDMVNPRPGTLRHAVIQKEPLWIVFKHDMSIRLSQELMITSDKTIDARGANVHIAYGAGITMQYVHNIIIHGLHVHHIVKSSGGLIRDSINHFGHRGEADGDGISIFGATNIWLDHISMSKCQDGLIDAIMGSTAITISNSHFTHHNDVMLLGAQNNNMDDKKMQVTVAYNHFGKGLVQRMPRVRWGFVHVVNNDYTHWELYAIGGSQGPTILSHGNRFIAPPHKQHYREVTKRDYASESEWKNWNWRSEKDVFMNNAYFRQSGNPHFKCSHSRQQMIKPKNGMAVSKLTKYAGALDCRVGKAC

> AT5G15110 (PLL 11)

MEMVRLSKLMFTFCIAVLIPTIRGNISELDEYWSQRADEAREFTLQAYHSDPYEIVDHFHERHYDNSTDVTTPEEDGDAKPEEEEKEFIEMLGSSTNSTRRSLRGKGKGKWSKLKGPCTASNPIDKCWRCRSDWAKRRKKLTRCVRGFGHRTTGGKRGRIYVVTSNLDEDMVNPKPGTLRHAVIQKEPLWIIFKNDMSIRLNQELLINSHKTIDARGANVHVAHGAGITMQFVKNVVIHGLHIHHISESSGGMIRDSVDHFGMRTRADGDGLSIYGSSNIWLDHISMSKCQDGLIDAIVGSTGITISNSHFTHHNDVMLLGAQNTNEADKHMQVTVAYNHFGKGLVQRMPRIRWGFVHVVNNDYTHWELYAIGGSQGPTILSHGNRFIAPPHKPHYREVTKRDYASEDEWKHWNWRSDKDVFMNGAYFRQSGNPQYKCAHTRQQMIKPKNGLAVSKLTKYAGALDCRVGRRC

> AT5G04310 (PLL 12)

MVAHERRIHNLQKPTCICIIWFCLLVSLSHHGRASSTSASIFNLSLPHQHPFPEHVVLNVQRKLNDSLSRRQLLTYQQDDGTTASSPIPSCITGNPIDDCWRCDPNWSANRQRLADCSIGFGQGTLGGKGGQFYLVTDSSDNDAANPIPGTLRHAVIQPEPLWIIFSSDMGIKLKHELIIGSYKTIDGRGTNIQITGHGCLTIQQVSHVIIHNVHIHHCKPSGNTLVASSPTHVGFRGVSDGDGISVSASHHIWVDHCSLGYCADGLIDVILASTAVTISNNYFSHHDEVMLLGHDDRYTADKGMQVTIAFNHFGEGLVQRMPRCRHGYIHVVNNDFTAWEMYAIGGSASPTINSQGNRYTAPIDPNAKEVTKRVDSNEKHWSGWNWRTEGDVMVNGAFFVPSGDGVSPAYARATSVQPKAAAIIDQLTVNAGVFGDPSGRNGQGGSFPGITNGGGTITRGYSKSGPAGGGSGSDSDDGLFTLIFGNNSGAVALRPGQVWSILLIIILYWYIPHHTRS

> AT3G54920 (PLL 13)

MLLQNFSNTIFLLCLFFTLLSATKPLNLTLPHQHPSPDSVALHVIRSVNESLARRQLSSPSSSSSSSSSSSSSSCRTGNPIDDCWRCSDADWSTNRQRLADCSIGFGHGTLGGKNGKIYVVTDSSDNNPTNPTPGTLRYGVIQEEPLWIVFSSNMLIRLKQELIINSYKTLDGRGSAVHITGNGCLTLQYVQHIIIHNLHIYDCKPSAGFEKRGRSDGDGISIFGSQKIWVDHCSMSHCTDGLIDAVMGSTAITISNNYFTHHDEVMLLGHDDNYAPDTGMQVTIAFNHFGQGLVQRMPRCRRGYIHVVNNDFTEWKMYAIGGSGNPTINSQGNRYSAPSDPSAKEVTKRVDSKDDGEWSNWNWRTEGDLMENGAFFVASGEGMSSMYSKASSVDPKAASLVDQLTRNAGVFGGPRDDQGQSGNSYSPYGGDGGGGGSSGGSSGGGMDVMGGTTRGSSSSSGDDSNVFQMIFGSDAPSRPRLTLLFSLLMISVLSLSTLLL

> AT5G55720 (PLL 14)

MSIVCTFFLFLLNTSFAFAFAIPKPPIVRRLSTTVTSNSTASSCSANGNPIDECWRCDENWKDNRKNLADCAVGFGRDSIGGRAGEFYTVTDSGDDNPLNPTPGTLRYAATQDQPLWIIFDRDMVIQLKQDLQVASYKTIDGRGNNVQIAYGPCLTLYKVSNIIINNLYIHDCVPVKRNALSSLGGYSDGDGISIFESRDIWIDHCTLEKCYDGLIDAVNGSTDITISNSYMLNHNEVMLLGHSDEYSGDRDMRVTIAFNYFGEGLVQRMPRCRHGYFHIVNNIYRDWKMYAIGGSANPTIFSQGNVFIASNNQFTKEVTKRESADGDEEWKEWNWKSEGDEMVNGAFFTPSGKEDSPSYAKFSSMVARPASLLKTTHPSVGVLSCEIDQAC

> AT5G63180 (PLL 15)

MFRPNSLLIPSNLSTTKSQRNTMLNSSYLSFALIFFCCILFSALASSLPVSDPELVVEEVHRKINESISRRKLGFFSCGSGNPIDDCWRCDKDWEKNRKRLADCGIGFGKNAIGGRDGEIYVVTDPGNDDPVNPRPGTLRYAVIQDEPLWIIFKRDMTIQLKEELIMNSFKTLDGRGASVHISGGPCITIQYVTNIIIHGLHIHDCKQGGNTYVRDSPEHYGYRTVSDGDGVSIFGGSHVWVDHCSLSNCNDGLIDAIRGSTAITISNNYLTHHNKVMLLGHSDTYEQDKNMQVTIAFNHFGEGLVQRMPRCRHGYFHVVNNDYTHWEMYAIGGSANPTINSQGNRFLAPDDSSSKEVTKHEDAPEDEWRNWNWRSEGDLLLNGAFFTYSGAGPAKSSSYSKASSLAARPSSHVGEITIASGALSCKRGSHC

> AT1G67750 (PLL16)

MRMTLVHLSLSLFSCLLLVLSPTFIASTPVSEPELVVQEVNEKINASRRNLGVLSCGTGNPIDDCWRCDPKWEKNRQRLADCAIGFGKHAIGGRDGKIYVVTDSSDKDVVNPKPGTLRHAVIQDEPLWIIFARDMVIKLKEELIMNSFKTIDGRGASVHIAGGACITVQYVTNIIIHGVNIHDCKRKGNAYVRDSPSHYGWRTASDGDAVSIFGGSHVWVDHCSLSNCADGLIDAIHGSTAITISNNYLSHHNKVMLLGHSDSYTRDKNMQVTIAFNHFGEGLVQRMPRCRHGYFHVVNNDYTHWQMYAIGGSAAPTINSQGNRFLAPNDHVFKEVTKYEDAPRSKWKKWNWRSEGDLFLNGAFFTPSGGGASSSYAKASSLSARPSSLVASVTSNAGALFCRKGSRC

>AT3G53190 (PLL17)

MMLQRSCIVLFFSLFLLVPQMVFSMLNRTLLLIPHPDPELVAYQVQWKVNASITRRQALDTTDQAGSTPCITGNPIDDCWKCDPNWPNNRQGLADCGIGFGQYALGGKGGQFYFVTDSSDDDAVNPKPGTLRYGVIQEEPLWIVFPSNMMIKLKQELIFNSYKTLDGRGANVHIVGGGCITLQYVSNIIIHNIHIHHCYQSGNTNVRSSPTHYGFRTKSDGDGISIFGSKDIWIDHCSLSRCKDGLIDAVMGSTGITISNNFFSHHNEVMLLGHSDHYEPDSGMQVTIAFNHFGEKLIQRMPRCRRGYIHVVNNDFTQWEMYAIGGSGNPTINSQGNRYTAPTNPFAKEVTKRVETPDGDWKGWNWRSEGDILVNGAFFVASGEGAEMRYEKAYSVEPKSASFITQITFHSGVLGVGGRNNNLGMWTTTGSEGTSGLDSYNDYTDEMSGAGSTNRLSFSVLVFLLSSISYLVVFTSSTQMFML

> AT3G27400 (PLL 18)

MVSYSNNHFAYAFLLLLTIGNTLAFSSSLPDHVQDPNLVVDDVNRSVFNASRRSLAYLSCRTGNPIDDCWRCDPNWETNRQRLADCAIGFGKNAIGGRKGRIYVVTDPANDDPVNPRPGTLRYAVTQEEPLWIIFKRDMVIRLKKELIITSFKTIDGRGSSVHITDGPCLKIHYATNIIIHGINIHDCKPGSGGMIKDGPHHTGWWMQSDGDAVAIFGGKHVWIDHCSLSNCDDGLIDAIHGSTAITISNNHMTHHDKVMLLGHSDSYTQDKNMQVTIAFNHFGEGLVQRMPRCRHGYFHVVNNDYTHWEMYAIGGSASPTIYSQGNRFLAPNTRFNKEVTKHEDAPESKWRDWNWRSEGDMLLNGAYFRESGAEAPSTYARASSLSARPSSLVGSITTTAGTLSCRRGRRC

> AT4G24780 (PLL19)

MKMQTKKLFITIVSFLLYAPLFLSSPVPDPESVVEEVHKSINASVAGRRKLGYLSCTTGNPIDDCWRCDPHWEQHRQRLADCAIGFGKNAIGGRDGRIYVVTDSGNDNPVSPKPGTLRHAVVQDEPLWIIFQRDMTIQLKEELIMNSFKTIDGRGASVHISGGPCITIQYVTNIIIHGIHIHDCKQGGNAMVRSSPRHFGWRTISDGDGVSIFGGSHVWVDHCSFSNCEDGLIDAIMGSTAITLSNNHMTHHDKVMLLGHSDTYSRDKNMQVTIAFNHFGEGLVQRMPRCRHGYFHVVNNDYTHWEMYAIGGSANPTINSQGNRFLAPNIRFSKEVTKHEDAPESEWKRWNWRSSGDLLLNGAFFTPSGGAASSSYAKASSLGAKPSSLVGPLTSTSGALNCRKGSRC

>AT3G07010 (PLL20)

MAVTKLILFASALLLTALFIGVNASRSNETWHEHAVENPDEVAAMVDMSIRNSTERRRLGYFSCATGNPIDDCWRCDRKWQLRRKRLADCSIGFGRNAIGGRDGRFYVVTDPGDDDPVNPIPGTLRHAVIQDEPLWIIFKRDMVITLKQELIMNSFKTIDGRGVNVHIANGACLTIQYVTNIIVHGIHVHDCKPTGNAMVRSSPSHYGFRSMADGDAISIFGSSHIWIDHNSLSNCADGLVDAVMSSTAITVSNNFFTHHNEVMLLGHSDSYTRDKVMQVTIAYNHFGEGLIQRMPRCRHGYFHVVNNDYTHWEMYAIGGSAGPTINSQGNRFLAPVNPFAKEVTKREYTGESKWKHWNWRSEGDLFLNGAFFTRSGAGAGANYARASSLSAKSSSLVGTMTSYSGALNCRAGRRC

> AT5G48900 (PLL 21)

MAVTQILVVFASALLLSMFFTGVDSTRSNETWHEHAVENPEEVAAMVDMSIRNSTARRRLGYFSCSTGNPIDDCWRCDRRWQSRRKHLANCAIGFGRNAIGGRDGRYYVVSDPNDDNPVNPKPGTLRHAVIQEEPLWIVFKRDMVITLKEELIMNSFKTIDGRGVNVHIANGACITIQFVTNIIIHGIHIHDCRPTGNAMVRSSPSHYGWRTMADGDGISIFGSSHIWIDHNSLSNCADGLIDAVMASTAITISNNYFTHHNEVMLLGHSDTYTRDKVMQVTIAYNHFGEGLIQRMPRCRHGYFHVVNNDYTHWEMYAIGGSASPTINSQGNRYLAPRNRFAKEVTKRDYAGQWQWRHWNWRSEGDLFLNGAFFTRSGSGLGASYARASSLAAKSSSLVGVITYNAGALNCRGGRRC

> AT3G24670 (PLL 22)

MVIFSRSFLALSTTLIILALCINSSTMAQETEDLNSHSSSNSSTANKLPNDDGAWNEHAVKNPEEVAAMVDMKIKNSTERRRLGFFSCATGNPIDDCWRCDRNWHLRRKRLANCAIGFGRNAIGGRDGRYYVVTDPSDHDAVNPRPGTLRHAVIQDRPLWIVFKRDMVITLTQELIMNSFKTIDGRGVNVAIAGGACITIQYVTNIIIHGINVHDCRRTGNAMVRSSPSHYGWRTMADGDAISIFGSSHIWIDHNSLSNCADGLIDAIMGSTAITISNNYMTHHNEVMLMGHSDSYTRDKLMQVTIAYNHFGEGLIQRMPRCRHGYFHVVNNDYTHWVMYAIGGSANPTINSQGNRFLAPGNPFAKEVTKRVGSWQGEWKQWNWRSQGDLMLNGAYFTKSGAAAPASYARASSLGAKPASVVSMLTYSSGALKCRIGMRC

> AT4G13210 (PLL 23)

MVVARTLFSISATLIIFLALFLHVNAVQETREPKHESSRNTSTVDNLSDGEWHEHAVKDPEEIAAMVDMSIRNSTYRRKLGFFSSCSTGNPIDDCWRCDKKWHRRRKRLADCAIGFGRNAVGGRDGRYYIVTDPSDHDPVTPKPGTLRYAVIQDEPLWIVFKRDMVITLSQELIMNSFKTIDGRGVNVHIAGGACLTVQYVTNIIIHGINIHDCKRTGNAMVRSSESHYGWRTMADGDGISIFGSSHIWIDHNSLSSCADGLIDAIMGSTAITISNNYLTHHNEAILLGHTDSYTRDKMMQVTIAYNHFGEGLIQRMPRCRHGYFHVVNNDYTHWEMYAIGGSANPTINSQGNRFLAPGNRFAKEVTKRVGAGKGEWNNWNWRSQGDLMLNGAYFTSSGAGASANYARASSLAAKSSSLVGMLTSSSGALKCRIGTLC

> AT3G24230 (PLL 24)

MATSSLKLTSACFVLLFIFVGCVLTATNLRNNEISRSRKLKTEDSKSFNSSPMTTRLDGVVELNEHAVTDPDKVAHEVSNLIHMSEQNITARRKLGFFSCGNGNLIDDCWRCDRNWNKNRKHLADCGMGFGSKAFGGRNGSYYVVTDHSDDDVVNPKPGTLRHAVIQVEPLWIIFKRDMVIKLKQELIMNSFKTIDARGANVHIANGACITIQNITNVIVHGLHIHDCKRTGNVTVRSSPSQAGFRGTADGDAINIFGSSHIWIDHNSLSNCTDGLVDVVNGSTAITISNNHFTHHDEVMLLGHNDSYTRDKMMQVTVAYNHFGEGLIQRMPRCRHGYFHVVNNDYTHWKMYAIGGSANPTINSQGNRFAAPKNHSAKEVTKRLDTKGNEWMEWNWRSEKDLLVNGAFFTPSGEGASGDSQTLSLPAKPASMVDAITASAGALSCRRGKPCY

> AT4G13710 (PLL 25)

MASSSQKLISVCVAVLVVLALTAMIFRNSEISLSRKLKTEVIQSSNSSTMAAIRKLKTEEFQSLNSSTMAATRLDGEPQQQQHAVADDPDMVADEVAKLVQMSEQNRTARRKLGFFSCGTGNPIDDCWRCDRNWHKNRKRLADCGIGFGRNAIGGRDGRFYIVTDPTDEDVVNPKPGTLRHAVIQEEPLWIVFKRDMVIELKQELIMNSFKTIDARGSNVHIANGACITIQFITNVIIHGLHIHDCKPTGNAMVRSSPSHFGWRTMADGDAVSIFGSSHIWIDHNSLSHCADGLVDAVMGSTAITVSNNHFTHHNEVMLLGHSDSYTKDKLMQVTIAYNHFGEGLVQRMPRCRHGYFHVVNNDYTHWEMYAIGGSAEPTINSQGNRYAAPMDRFAKEVTKRVETDASEWKKWNWRSEGDLLLNGAFFRPSGAGASASYGRASSLAAKPSSMVDTITSTAGALGCRKGRPC

> AT1G04680 (PLL 26)

MAVLPTWLLAMMCLLFFVGAMENTTHDNISSLPRSDETEWNQHAVTNPDEVADEVLALTEMSVRNHTERRKLGYFTCGTGNPIDDCWRCDPNWHKNRKRLADCGIGFGRNAIGGRDGRFYVVTDPRDDNPVNPRPGTLRHAVIQDRPLWIVFKRDMVIQLKQELIVNSFKTIDGRGANVHIANGGCITIQFVTNVIVHGLHIHDCKPTGNAMVRSSETHFGWRTMADGDAISIFGSSHVWIDHNSLSHCADGLVDAVMGSTAITISNNHLTHHNEVMLLGHSDSYMRDKAMQVTIAYNHFGVGLIQRMPRCRHGYFHVVNNDYTHWEMYAIGGSANPTINSQGNRYAAPKNPFAKEVTKRVDTPASHWKGWNWRSEGDLLQNGAYFTSSGAAASGSYARASSLSAKSSSLVGHITSDAGALPCRRGRQCSS

>FaPEL ([AAK66161.1](https://www.ncbi.nlm.nih.gov/protein/14531296?report=genbank&log$=prottop&blast_rank=2&RID=6P6SDB6U014))

SIRNSTERRKLGYFSCGTGNPIDDCWRCDPNWQKNRKRLADCGIGFGRNAIGGRDGRFYVVTDPNDDDPVNPRPGTLRHAVIQDEPLWIVFKRDMVIQLKQELIMNSFKTIDGRGVNVHIANGACITIQFVTNVIVHGLHIHDCKPTGNAMVRSSPSHFGWRTMADGDAISIFGSSHIWVDHNSLSNCADGLVDAVMGSTAITISNNHLTHHNEVMLLGHSDSYTRDKQMQVTIAYNHFGEGLIQRMPRCRHGYFHVVNNDYTHWEMYAIGGSADPTINSQGNRYAAPTNPFAKEVTKRVETSQTQWRGWNWRSEGDLLLNGAFFTPSGAGASAVYARASSLGAKSSAMVGTITASAGALGCRRGRTC

>MaPEL ([AAF19196.1](https://www.ncbi.nlm.nih.gov/protein/6606534?report=genbank&log$=prottop&blast_rank=1&RID=6P6WVY6G014))

MTAGLRWIPPLLLLLLGFLLVLNGSRGWIGSERSSGSRNGGASRRSLREASANATSADASLEERAVTRAAEAAVDDPEEVASTVLTTIINSTARRSLGYLSCGSGNPIDDCWRCDPDWHVNRKKLADCGIGFGRNAIGGRDGELYVVTDSGDDDPVNPRPGTLRYAVIQDVPLWITFKHDMEITLKEELIMNSFKTIDGRGVNVHIANGACITIQYITNVIIHGLHIHDCKPTGNAMVRSSPSHYGWRTMADGDAVSIFGSSHIWVDHCSLSNCADGLVDAVMGSTAITVSNNYFTHHNEVMLLGHTDSYARDSIMQVTIAFNHFGEGLIQRMPRCRHGYFHVVNNDYTHWEMYAIGGSANPTINSQGNRYLAPTNPFAKEVTKRVDTDQSTWKNWNWRSEGDLLLNGAFFTPSGAGASASYARASSFGAKPSSLVDTLTSDAGVLSCQVGTRC
